# Clinical Characterization and Prediction of Clinical Severity of SARS-CoV-2 Infection Among US Adults Using Data From the US National COVID Cohort Collaborative

**DOI:** 10.1001/jamanetworkopen.2021.16901

**Published:** 2021-07-13

**Authors:** Tellen D. Bennett, Richard A. Moffitt, Janos G. Hajagos, Benjamin Amor, Adit Anand, Mark M. Bissell, Katie Rebecca Bradwell, Carolyn Bremer, James Brian Byrd, Alina Denham, Peter E. DeWitt, Davera Gabriel, Brian T. Garibaldi, Andrew T. Girvin, Justin Guinney, Elaine L. Hill, Stephanie S. Hong, Hunter Jimenez, Ramakanth Kavuluru, Kristin Kostka, Harold P. Lehmann, Eli Levitt, Sandeep K. Mallipattu, Amin Manna, Julie A. McMurry, Michele Morris, John Muschelli, Andrew J. Neumann, Matvey B. Palchuk, Emily R. Pfaff, Zhenglong Qian, Nabeel Qureshi, Seth Russell, Heidi Spratt, Anita Walden, Andrew E. Williams, Jacob T. Wooldridge, Yun Jae Yoo, Xiaohan Tanner Zhang, Richard L. Zhu, Christopher P. Austin, Joel H. Saltz, Ken R. Gersing, Melissa A. Haendel, Christopher G. Chute

**Affiliations:** 1 Section of Informatics and Data Science, Department of Pediatrics, University of Colorado School of Medicine, University of Colorado, Aurora; 2 Department of Biomedical Informatics, Stony Brook University, Stony Brook, New York; 3 Stony Brook University, Stony Brook, New York; 4 Palantir Technologies, Denver, Colorado; 5 Department of Internal Medicine, The University of Michigan at Ann Arbor, Ann Arbor; 6 Department of Public Health Sciences, University of Rochester Medical Center, Rochester, New York; 7 Institute for Clinical and Translational Research, Johns Hopkins University School of Medicine, Baltimore, Maryland; 8 Department of Medicine, Johns Hopkins University School of Medicine, Baltimore, Maryland; 9 Sage Bionetworks, Seattle, Washington; 10 Department of Medicine, Johns Hopkins University School of Medicine, Baltimore, Maryland; 11 Division of Biomedical Informatics, Department of Internal Medicine, University of Kentucky, Lexington; 12 Real World Solutions, IQVIA, Cambridge, Massachusetts; 13 Observational Health Data Sciences and Informatics, New York, New York; 14 Division of Health Science Informatics, Department of Medicine, Johns Hopkins University School of Medicine, Baltimore, Maryland; 15 Department of Orthopaedic Surgery, University of Alabama at Birmingham, Birmingham; 16 Translational and Integrative Sciences Center, Oregon State University, Corvallis; 17 Department of Biomedical Informatics, University of Pittsburgh, Pittsburgh, Pennsylvania; 18 Department of Biostatistics, Johns Hopkins University School of Medicine, Baltimore, Maryland; 19 TriNetX, Cambridge, Massachusetts; 20 North Carolina Translational and Clinical Sciences Institute, University of North Carolina at Chapel Hill, Chapel Hill; 21 Department of biomedical informatics, Stony Brook University, Stony Brook, New York; 22 Department of Preventive Medicine and Public Health, University of Texas Medical Branch, Galveston; 23 Oregon Clinical and Translational Research Institute, Oregon Health & Science University, Portland; 24 Tufts Medical Center Clinical and Translational Science Institute, Tufts Medical Center, Boston, Massachusetts; 25 National Center for Advancing Translational Sciences, National Institutes of Health, Bethesda, Maryland; 26 Center for Health AI, University of Colorado, Aurora; 27 Department of Health Policy and Management, Johns Hopkins University School of Medicine, Baltimore, Maryland; 28 Department of Medicine, Johns Hopkins University School of Medicine, Baltimore, Maryland; 29 Department of Nursing, Johns Hopkins University School of Medicine, Baltimore, Maryland

## Abstract

**Importance:**

The National COVID Cohort Collaborative (N3C) is a centralized, harmonized, high-granularity electronic health record repository that is the largest, most representative COVID-19 cohort to date. This multicenter data set can support robust evidence-based development of predictive and diagnostic tools and inform clinical care and policy.

**Objectives:**

To evaluate COVID-19 severity and risk factors over time and assess the use of machine learning to predict clinical severity.

**Design, Setting, and Participants:**

In a retrospective cohort study of 1 926 526 US adults with SARS-CoV-2 infection (polymerase chain reaction >99% or antigen <1%) and adult patients without SARS-CoV-2 infection who served as controls from 34 medical centers nationwide between January 1, 2020, and December 7, 2020, patients were stratified using a World Health Organization COVID-19 severity scale and demographic characteristics. Differences between groups over time were evaluated using multivariable logistic regression. Random forest and XGBoost models were used to predict severe clinical course (death, discharge to hospice, invasive ventilatory support, or extracorporeal membrane oxygenation).

**Main Outcomes and Measures:**

Patient demographic characteristics and COVID-19 severity using the World Health Organization COVID-19 severity scale and differences between groups over time using multivariable logistic regression.

**Results:**

The cohort included 174 568 adults who tested positive for SARS-CoV-2 (mean [SD] age, 44.4 [18.6] years; 53.2% female) and 1 133 848 adult controls who tested negative for SARS-CoV-2 (mean [SD] age, 49.5 [19.2] years; 57.1% female). Of the 174 568 adults with SARS-CoV-2, 32 472 (18.6%) were hospitalized, and 6565 (20.2%) of those had a severe clinical course (invasive ventilatory support, extracorporeal membrane oxygenation, death, or discharge to hospice). Of the hospitalized patients, mortality was 11.6% overall and decreased from 16.4% in March to April 2020 to 8.6% in September to October 2020 (*P* = .002 for monthly trend). Using 64 inputs available on the first hospital day, this study predicted a severe clinical course using random forest and XGBoost models (area under the receiver operating curve = 0.87 for both) that were stable over time. The factor most strongly associated with clinical severity was pH; this result was consistent across machine learning methods. In a separate multivariable logistic regression model built for inference, age (odds ratio [OR], 1.03 per year; 95% CI, 1.03-1.04), male sex (OR, 1.60; 95% CI, 1.51-1.69), liver disease (OR, 1.20; 95% CI, 1.08-1.34), dementia (OR, 1.26; 95% CI, 1.13-1.41), African American (OR, 1.12; 95% CI, 1.05-1.20) and Asian (OR, 1.33; 95% CI, 1.12-1.57) race, and obesity (OR, 1.36; 95% CI, 1.27-1.46) were independently associated with higher clinical severity.

**Conclusions and Relevance:**

This cohort study found that COVID-19 mortality decreased over time during 2020 and that patient demographic characteristics and comorbidities were associated with higher clinical severity. The machine learning models accurately predicted ultimate clinical severity using commonly collected clinical data from the first 24 hours of a hospital admission.

## Key Points

**Question:**

In a US data resource large enough to adjust for multiple confounders, what risk factors are associated with COVID-19 severity and severity trajectory over time, and can machine learning models predict clinical severity?

**Findings:**

In this cohort study of 174 568 adults with SARS-CoV-2, 32 472 (18.6%) were hospitalized and 6565 (20.2%) were severely ill, and first-day machine learning models accurately predicted clinical severity. Mortality was 11.6% overall and decreased from 16.4% in March to April 2020 to 8.6% in September to October 2020.

**Meaning:**

These findings suggest that machine learning models can be used to predict COVID-19 clinical severity with the use of an available large-scale US COVID-19 data resource.

## Teaser

This cohort study evaluates COVID-19 severity and factors associated with severity over time and assesses the use of machine learning to predict clinical severity.

## Introduction

As of the middle of December 2020, SARS-CoV-2 had infected more than 70 million people and caused more than 1.6 million deaths worldwide.^1^ SARS-CoV-2 can cause COVID-19, a condition characterized by pneumonia, hyperinflammation, hypoxemic respiratory failure, a prothrombotic state, cardiac dysfunction, substantial mortality, and persistent morbidity in some survivors. Few therapeutic interventions authorized by the US Food and Drug Administration are available, and vaccine deployment has been slow. Progress in COVID-19 research has been slowed by a lack of broad access to clinical data. Investigators in the United Kingdom^2^ and Denmark^2,3^ have performed person-level analytical analyses across their respective populations to inform health care delivery, medication decisions, and national interventions, but the US has not had this capacity. A large, multicenter, representative clinical data set has been desperately needed by US practitioners, scientists, health care systems, and policymakers to develop predictive and diagnostic computational tools and to inform critical decisions.

To address these gaps, the National COVID Cohort Collaborative (N3C) was formed to accelerate understanding of SARS-CoV-2 and develop a novel approach for collaborative data sharing and analytical data during the pandemic. The N3C^4^ is composed of members from the National Institutes of Health Clinical and Translational Science Awards Program and its Center for Data to Health, the IDeA Centers for Translational Research,^5^ the National Patient-Centered Clinical Research Network, the Observational Health Data Sciences and Informatics network, TriNetX, and the Accrual to Clinical Trials network.

This report provides a detailed clinical description of the largest cohort of US COVID-19 cases and representative controls to date. This cohort is racially and ethnically diverse and geographically distributed. We evaluated COVID-19 severity and associated clinical and demographic factors over time and used machine learning to develop a clinically useful model that accurately predicts severity using data from the first day of hospital admission.

## Methods

### Regulatory Approvals

The N3C Data Enclave is approved under the authority of the National Institutes of Health Institutional Review Board. Each N3C site maintains an institutional review board–approved data transfer agreement. The analyses reported in this article were approved separately by the institutional review board of each institution of investigators with data access. This approval included a waiver of informed consent. Data were not deidentified. See the eMethods in Supplement 1 for details about each level of regulatory approval.

### Cohort Definition and Outcome Stratification

Because of the broad inclusion criteria, the N3C includes cases and appropriate controls for varied analyses, including both outpatients and inpatients (eMethods and eTable 1 in Supplement 1). The N3C includes patients with any encounter after January 1, 2020, with (1) 1 of a set of a priori–defined SARS-CoV-2 laboratory tests, or (2) a strong positive diagnostic code, or (3) 2 weak positive diagnostic codes during the same encounter or on the same date before May 1, 2020. The cohort definition is publicly available on GitHub.^6^ For the N3C patients, encounters in the same health care system beginning on or after January 1, 2018, are also included to provide information about preexisting health conditions. See eMethods in Supplement 1 for information about the N3C architecture, data ingestion, and integration.

**Figure 1.**
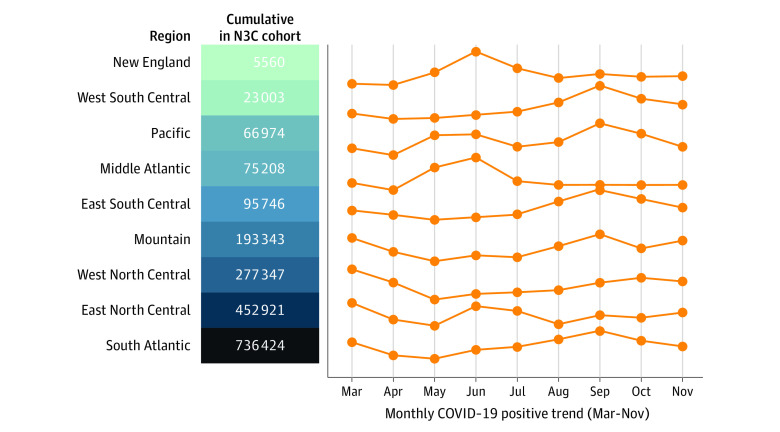
Geographic Distribution of Overall SARS-CoV-2–Positive Patients in the US National COVID Cohort Collaborative (N3C) Cohort (N = 1 926 526) Trend lines show the accumulation of each subregion’s sample size of laboratory-confirmed positive cases in 2020. The Southeast, Middle Atlantic, and Midwestern regions are the most heavily represented, but all regions have substantial patient counts.

We conducted a retrospective cohort study of adults 18 years or older at the 34 N3C sites whose data had completed harmonization and integration (eMethods in Supplement 1) and were released for analysis on December 7, 2020, and included the necessary death and mechanical ventilatory support information (eFigure 1 in Supplement 1). Initial analyses (Figure 1; eTable 1 in Supplement 1) are based on the entire cohort to demonstrate the scope of the N3C. All subsequent analyses include only patients with a SARS-CoV-2 laboratory test (polymerase chain reaction [PCR] or antigen) (Table). We then performed analyses on those with positive test results and hospitalized patients.

**Table.** Characteristics and Clinical Course of the Patients With SARS-CoV-2^a^

Characteristic	Outpatients with mild conditions (WHO severity, 1-3) (n = 121 078)	Outpatients with mild conditions with ED visits (WHO severity, approximately 3) (n = 21 018)	Hospitalized patients with moderate condition without invasive ventilatory assistance (WHO severity, 4-6) (n = 25 907)	Hospitalized patients with severe conditions with invasive ventilatory support or ECMO (WHO severity, 7-9) (n = 2790)	Patients who died or were discharged to hospice (WHO severity, 10) (n = 3775)
Age, mean (SD), y	41.1 (17.2)	43.4 (16.8)	55.0 (19.1)	57.0 (15.4)	71.8 (14.7)
Sex					
Female	65 435 (54.0)	11 410 (54.3)	13 396 (51.7)	1089 (39.0)	1564 (41.4)
Male	55 526 (45.9)	9605 (45.7)	12 506 (48.3)	1697 (60.8)	2211 (58.6)
Other^b^	117 (0.1)	≤20^c^	≤20	≤20	0
Race					
White	70 330 (58.1)	7786 (37.0)	10 739 (41.5)	1020 (36.6)	1912 (50.6)
Black or African American	14 616 (12.1)	6351 (30.2)	8003 (30.9)	869 (31.1)	1101 (29.2)
Native Hawaiian or Pacific Islander	267 (0.2)	40 (0.2)	66 (0.3)	≤20	≤20
Asian	2778 (2.3)	564 (2.7)	717 (2.8)	86 (3.1)	120 (3.2)
Other	1030 (0.9)	403 (1.9)	373 (1.4)	51 (1.8)	48 (1.3)
Missing or unknown	32 057 (26.5)	5874 (27.9)	6009 (23.2)	757 (27.1)	584 (15.5)
Ethnicity					
Hispanic	18 539 (15.3)	5312 (25.3)	5145 (19.9)	610 (21.9)	476 (12.6)
Non-Hispanic	80 188 (66.2)	12 510 (59.5)	17 313 (66.8)	1789 (64.1)	2779 (73.6)
Missing or unknown	22 351 (18.5)	3196 (15.2)	3449 (13.3)	391 (14.0)	520 (13.8)
Insurance payer					
Medicare	2480 (2.0)	906 (4.3)	2852 (11.0)	308 (11.0)	823 (21.8)
Commercial	11 718 (9.7)	2277 (10.8)	1984 (7.7)	227 (8.1)	237 (6.3)
Medicaid	2945 (2.4)	1590 (7.6)	1974 (7.6)	242 (8.7)	294 (7.8)
Other	115 480 (95.4)	18 576 (88.4)	22 876 (88.3)	2409 (86.3)	3124 (82.8)
BMI, mean (SD)	30.1 (7.6) (n = 39 836)	31.2 (7.8) (n = 9552)	31.0 (9.0) (n = 16 489)	32.9 (9.4) (n = 1862)	29.5 (8.7) (n = 2440)
Weight, mean (SD), kg	86.3 (23.7) (n = 47 284)	87.3 (23.7) (n = 13 511)	88.6 (26.0) (n = 20 068)	95.5 (26.8) (n = 2349)	84.6 (26.7) (n = 3106)
Hospital LOS, median (IQR), d	NR	NR	4 (2-8) (n = 25 906)	23 (12-37) (n = 2790)	9 (4-18) (n = 3775)

Abbreviations: BMI, body mass index (calculated as weight in kilograms divided by height in meters squared); ECMO, extracorporeal membrane oxygenation; ED, emergency department; IQR, interquartile range; LOS, length of stay; NR, not reported; WHO, World Health Organization.

^a^ Data are presented as number (percentage) of patients unless otherwise indicated. Patients were stratified using the Clinical Progression Scale established by the World Health Organization for COVID-19 clinical research.^7^ Severity was assigned by patient-specific encounter maximum severity.

^b^ Other includes nonbinary, no matching concept, and no information.

^c^ Per National COVID Cohort Collaborative policy, we censored any cells with 1 to 20 patients and replaced them with 20 or fewer.

### Hospital Index Encounter and Clinical Severity

We defined a single index encounter for each patient with laboratory-confirmed SARS-CoV-2 using a prespecified algorithm (eMethods in Supplement 1). We stratified patients using the Clinical Progression Scale (CPS) established by the World Health Organization (WHO) for COVID-19 clinical research.^7^ We placed patients with positive test results into strata defined by the maximum clinical severity during their index encounter (Table). We collapsed some WHO CPS categories because of data limitations (eg, some sites do not submit fraction of inspired oxygen).

### Variable Definition

We defined or identified existing concept sets in the Observational Medical Outcomes Partnership common data model (CDM) for each clinical concept (eg, laboratory measure, vital sign, or medication) (eMethods in Supplement 1). We validated each concept set with input from informatics and clinical subject matter experts. Race/ethnicity were evaluated because of hypothesized and previously reported associations between race/ethnicity and COVID-19 outcomes. Race/ethnicity were defined during clinical care at each N3C site. All concept sets and analytic pipelines are fully reproducible and will be made publicly available.

### Statistical Analysis

We tested time trends using linear regression and differences between groups using multivariable logistic regression. We used 2-tailed, unpaired *t* tests to assess for differences in clinical and demographic characteristics between hospitalized and nonhospitalized patients with SARS-CoV-2 and 1-way analysis of variance (ANOVA) at day 7 to test for differences in biomarker trajectories (laboratory findings and vital signs) between severity groups. Statistical significance was defined as α ≤ .05; no multiple-testing corrections were made. We developed models to predict patient-specific maximum clinical severity: hospitalization with death, discharge to hospice, invasive mechanical ventilatory support, or extracorporeal membrane oxygenation (ECMO) vs hospitalization without any of those. To avoid immortal time bias, we only included patients with at least 1 hospital overnight (90.5% of inpatients). We split the hospitalized patients with laboratory-confirmed SARS-CoV-2 into randomly selected 70% training and 30% testing cohorts stratified by outcome proportions and held out the testing set. We chose a broad set of potential predictors present for at least 15% of the training set (eTable 2 in Supplement 1). The input variables are the most abnormal value on the first calendar day of the hospital encounter. When patients did not have a laboratory test value on the first calendar day, we imputed normal values for specialized laboratory tests (eg, ferritin and procalcitonin) and the median cohort value for common laboratory tests (eg, sodium and albumin) (eTable 2 in Supplement 1). We compared several analytical approaches with varying flexibility and interpretability: logistic regression with or without L1 and L2 penalty, random forest, support vector machines, and XGBoost (eMethods in Supplement 1). We internally validated models and limited overfitting using 5-fold cross-validation and evaluated models using the testing set and area under the receiver operator characteristic (AUROC) as the primary metric. Secondary metrics included precision (positive predictive value), recall (sensitivity), balanced accuracy, and F1 measure. Because SARS-CoV-2 outcomes have improved over time,^8^ we evaluated model performance overall and for March to May 2020 and June to October 2020. See eMethods in Supplement 1 for additional information including software packages used.

## Results

### Study Cohort

As of December 7, 2020, data from 34 sites were harmonized and integrated into the N3C release set. On that date, the N3C database included data from 1 926 526 patients (eTable 1 in Supplement 1). The patients derive from all US geographic regions but are more concentrated in the Southeast, Middle Atlantic, and Midwest (Figure 1). The age, sex, race/ethnicity, and insurance payer distributions (eFigure 2 and eTable 1 in Supplement 1) indicate a diverse patient cohort that is representative of many segments of the US population. Of importance, African American and Hispanic patients, who have disproportionately had COVID-19,^9^ are represented in sufficient numbers to support robust subgroup analyses, pathophysiologic hypothesis generation, and testing of algorithms and models to avoid bias (Table). eTable 3 in Supplement 1 reports the cohort findings stratified by CDM and strengths and weaknesses of each CDM. Figure 1 shows cohort geographic distribution evolution during 2020.

The study cohort included 174 568 adults (9.1% of overall) (mean [SD] age, 44.4 [18.6] years; 53.2% female) who tested positive for SARS-CoV-2 at a site with death and ventilatory support data available (Table). Antigen tests represent less than 5% of a single site’s positive test results. All other patients who tested positive for SARS-CoV-2 had positive PCR test results. We compared these patients with 1 133 848 controls who tested negative for SARS-CoV-2 at those sites (mean [SD] age, 49.5 [19.2] years; 57.1% female).

### Clinical Course and Mortality

Of the 174 568 adults with SARS-CoV-2, 32 472 (18.6%) were hospitalized, and 6565 (20.2%) of those had a severe clinical course (invasive ventilation, ECMO, death, or discharge to hospice). The median length of hospital stay was 5 days (interquartile range, 2-10), and 29 383 patients (90.5%) stayed overnight at least 1 night. Mortality (including discharge to hospice) was 11.6% among hospitalized patients (Table). Others^10^ have reported that inpatient mortality has decreased over time. We confirm this finding in our study: inpatient mortality decreased from 16.4% in March and April to 8.6% in September and October (*F* test for monthly linear trend *P* = .002). Our data also indicate that clinical severity has shifted toward less invasive mechanical ventilatory support and/or ECMO as the pandemic has progressed.

### Demographic Characteristics, Comorbidities, and Obesity

**Figure 2.**
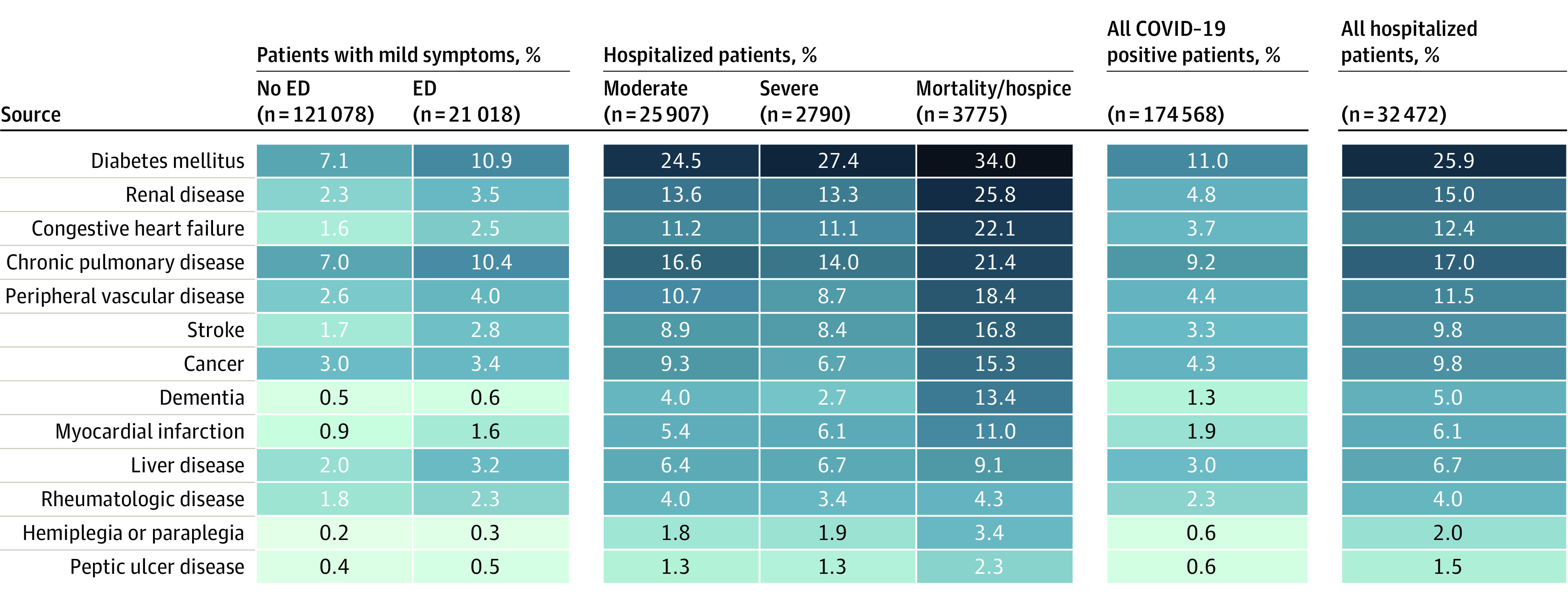
Comorbidity Distributions of the SARS-CoV-2–Positive Cohort (N = 174 568) See eMethods in Supplement 1 for comorbidity definitions. Patients were stratified using the Clinical Progression Scale (CPS) established by the World Health Organization (WHO) for COVID-19 clinical research (Table).^7^ Severity assigned by patient-specific encounter maximum severity. No ED indicates outpatient only without emergency department visit; ED, emergency department visit; moderate, hospitalized without invasive ventilatory support or extracorporeal membrane oxygenation (ECMO); severe, hospitalized with invasive ventilatory support or ECMO; mortality/hospice, hospital death or discharge to hospice.

**Figure 3.**
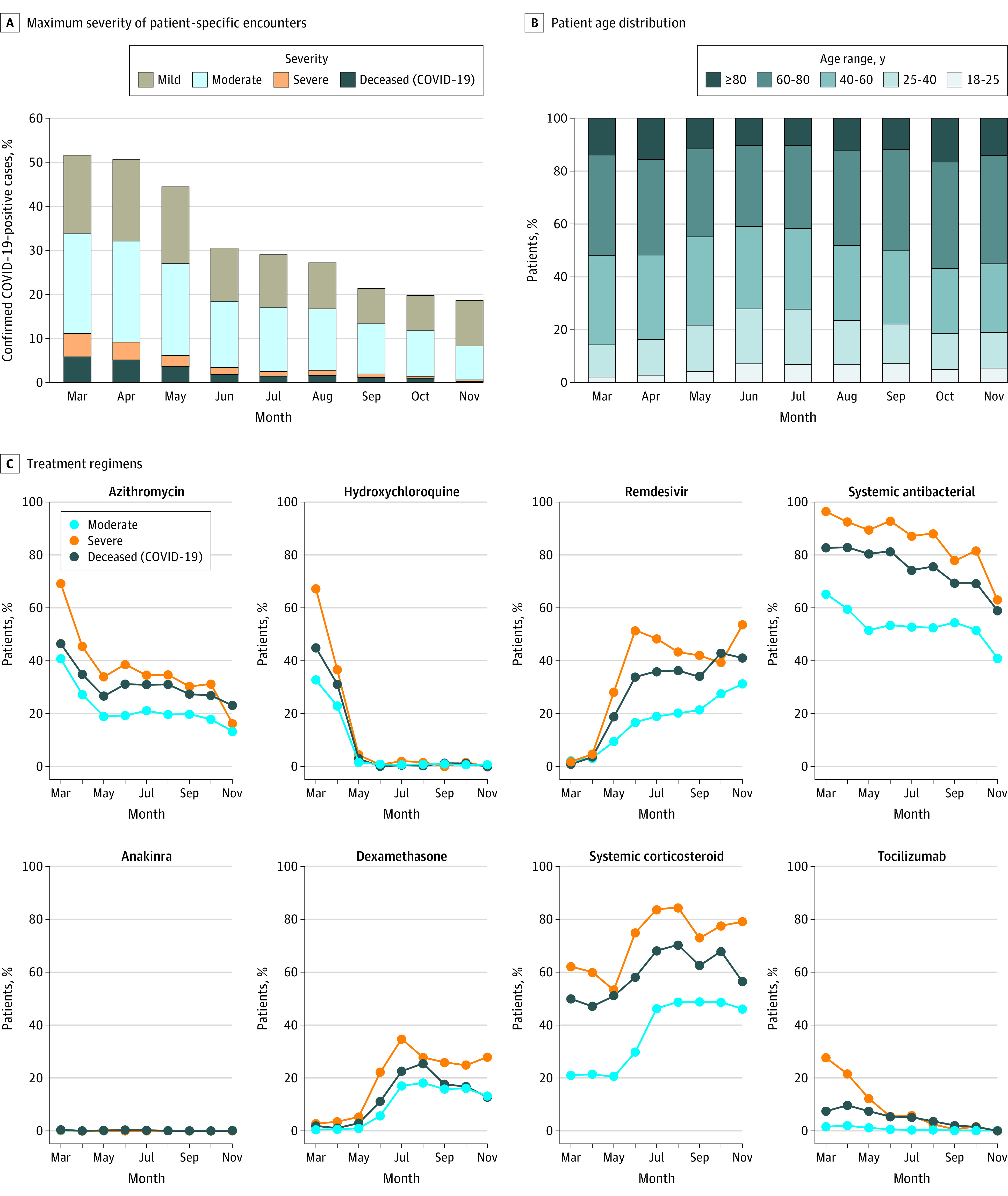
Clinical Severity, Age, and Antimicrobial and Immunomodulatory Medication Use Over Time A, Distribution of the maximum severity of patient-specific encounter among hospitalized patients during 2020. Mortality and invasive ventilatory assistance or extracorporeal membrane oxygenation (severe) have decreased steadily (monthly trend *P* = .002). Strata were assigned using the Clinical Progression Scale (CPS) established by the World Health Organization (WHO) for COVID-19 clinical research (Table).^7^ B, Age distribution of hospitalized patients during 2020. Older patients were more prominent in the spring and the fall, with more younger patients in the summer. C, Evolution of antimicrobial (top) and immunomodulatory (bottom) treatment regimens for hospitalized patients (top 3 severity strata [Table]) during 2020.

Data on preexisting health conditions that allowed calculation of comorbidities were present for 49% of hospitalized patients. Of hospitalized patients, 41% had at least 1 comorbid condition; the most common was diabetes (25.9%) (Figure 2). Mean body mass index (calculated as weight in kilograms divided by height in meters squared) was 30 or above (indicating obesity) for all severity groups except hospital death or discharge to hospice (29.5, indicating overweight) (Table). The age distribution for hospitalized patients was older during spring 2020, younger during the summer, and older again in the fall (Figure 3). In a multivariable logistic regression model built for inference, age (odds ratio [OR], 1.034 per year; 95% CI, 1.032-1.036), male sex (OR, 1.60; 95% CI, 1.51-1.69), liver disease (OR, 1.20; 95% CI, 1.08-1.34), dementia (OR, 1.26; 95% CI, 1.13-1.41), African American (OR, 1.12; 95% CI, 1.05-1.20) and Asian (OR, 1.33; 95% CI, 1.12-1.57) race, and obesity (body mass index >30; OR, 1.36; 95% CI, 1.27-1.46) were independently associated with higher clinical severity (invasive ventilatory support, ECMO, death, or discharge to hospice vs none of those) (eTable 4 in Supplement 1). Of interest, rheumatologic disease and blood type AB had protective associations. This analysis was conducted only to provide inference about previously reported risk factors and occurred after the prediction model was built.

### Vital Sign and Laboratory Measurements

**Figure 4.**
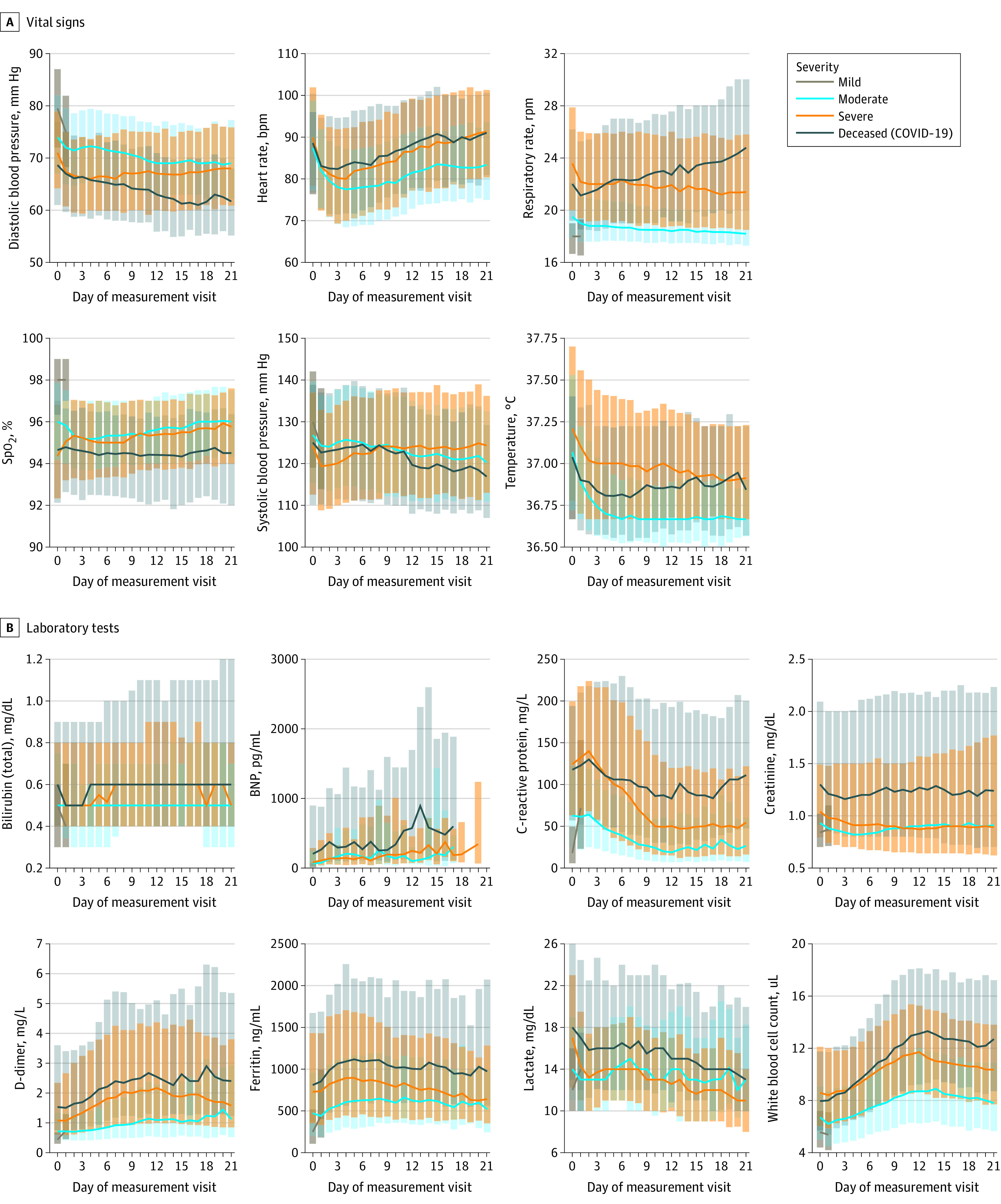
Trajectories of Vital Signs and Laboratory Tests During a Hospital Encounter A, Medians (line) and interquartile ranges (error bars) of each vital sign on each hospital day, stratified by patient maximum severity (Table). B, Medians (line) and interquartile ranges (error bars) of each laboratory test on each hospital day, stratified by the same severity groups. We tested trajectory differences between severity groups using 1-way analysis of variance at day 7. BNP indicates brain-type natriuretic peptide; Spo_2_, saturation as measured by pulse oximetry. SI conversion factors: To convert bilirubin to micromoles per liter, multiply by 17.104; BNP to nanograms per liter, multiply by 1; C-reactive protein to milligrams per liter, multiply by 10; creatinine to micromoles per liter, multiply by 88.4; D-dimer to nanomoles per liter, multiply by 5.476; ferritin to micrograms per liter, multiply by 1; lactate to millimoles per liter, multiply by 0.111; and white blood cells to ×10^9^/L, multiply by 0.001.

As a hospital encounter progressed, those who ultimately developed higher clinical severity (invasive ventilatory support, ECMO, or death) tended to have progressively more abnormal (higher) mean heart rate, respiratory rate, and temperature than those who did not (Figure 4A). Mean diastolic blood pressure and oxygen saturation among those who ultimately died continued to have more abnormal (lower) values, whereas those who received invasive ventilatory support or ECMO had more normal (higher) values (Figure 4A). Early in the hospital encounter, mean values of diastolic blood pressure, oxygen saturation, and widely used measures of inflammation (C-reactive protein and ferritin), immunologic activation (white blood cell count), fibrinolysis (D-dimer), oxygen delivery (lactate), and kidney function (creatinine) were more abnormal among those who ultimately required invasive ventilatory assistance or ECMO than those who did not (Figure 4A and B). These findings support the hypothesis that clinical severity can be predicted using information available early in a hospital course (see prediction models).

Other measurements (eg, sodium, platelet count, and lymphocyte count) have potential utility as early outcome predictors because their values near the beginning of a hospital encounter tend to separate patients with lower and higher maximum clinical severity (eFigure 3 in Supplement 1). Mean values of brain-type natriuretic peptide were low early in hospital encounters but had meaningful spikes between hospital days 10 and 15. This finding is consistent with a previous report^11^ of the timing of cardiac failure in COVID-19. Overall, patients with more abnormal nadir and/or peak values of several vital signs and laboratory measurements were more often represented in higher severity groups (invasive ventilatory support, ECMO, or death) (eFigure 4A and B in Supplement 1). C-reactive protein, ferritin, D-dimer, white blood cells, and interleukin 6 have been identified by the WHO as key biochemical parameters for a core COVID-19 outcome set.^7^ These values were measured in 44% to 94% of hospitalized patients, except interleukin 6 (7.6%). Only 9.1%, of hospitalized patients had blood type data (eFigure 5 in Supplement 1).

### Treatments

Use of antimicrobial and immunomodulatory medications has changed markedly over time (Figure 3C). Overall, 66.2% of the hospitalized cohort received at least 1 antimicrobial, with significant treatment regimen heterogeneity (eFigure 6A and eTable 5 in Supplement 1). Patients who received invasive ventilatory support and ECMO received more antimicrobials overall (eFigure 5A in Supplement 1). Antivirals with potential activity against SARS-CoV-2 were given to 16.7% (remdesivir) and 0.6% (lopinavir or ritonavir) of hospitalized patients. At least 1 immunomodulatory medication was given to 41.5% of hospitalized patients, also with wide variation in treatment regimen (eFigure 6B and eTable 5 in Supplement 1). More patients received hydrocortisone, methylprednisolone, and prednisone than dexamethasone (eTable 5A in Supplement 1). The trial that indicated a survival benefit from dexamethasone was published in July 2020.^12^ Another corticosteroid, hydrocortisone, also has modestly supportive clinical trial data.^13^

Of the hospitalized cohort, 14.0% received any invasive respiratory support (mechanical ventilatory support or inhaled or systemic pulmonary vasodilators) (eTable 5 in Supplement 1). Similarly, 8.3% received medications for cardiovascular support or ECMO, and 3.2% received dialysis or continuous renal replacement therapy.

### Severity Prediction

We developed several models that accurately predict a severe clinical course using data from the first hospital calendar day (eFigure 7 and eTable 6 in Supplement 1). The models with the best discrimination of severe vs nonsevere clinical course were built using XGBoost and random forest (AUROC = 0.87; 95% CI, 0.86-0.88 for both).^14^ Both are flexible, nonlinear, tree-based models that provide interpretability with a variable importance metric (eFigure 8 in Supplement 1). Of importance, discrimination by the 2 models was stable over time (March to May 2020 and June to October 2020) (eTable 6 in Supplement 1). This finding indicates that the models did not train on health care processes only typical during the pandemic’s chaotic first wave. Discrimination varied by region (AUROC = 0.78-0.95 over the 9 regions shown in Figure 1) but remained good in each. Commonly collected variables (age, oxygen saturation, respiratory rate, blood urea nitrogen, systolic blood pressure, and aspartate aminotransferase) were among the inputs with the highest variable importance for both models (eFigure 8 in Supplement 1).

## Discussion

This cohort study characterizes the largest US COVID-19 cohort to date, including 174 568 adults who tested positive for SARS-CoV-2. This study found a month-over-month decrease in COVID-19 inpatient mortality and invasive ventilatory support rates since March 2020, as well as striking changes in treatment patterns over time. The study also established expected trajectories for many vital signs and laboratory values among patients with different clinical severities. Expected trajectories can contribute to practitioner decision-making about what a patient will need.

Site heterogeneity in the distribution of predictors of severe COVID-19 disease, including age, race/ethnicity, and existing comorbidities (eg, diabetes), has complicated interpretation of their independent impact on outcomes. Like other studies, this study found that age, male sex,^2^ African American race,^9,15^ and obesity^16,17^ were associated with greater clinical severity. Associations of liver disease and dementia with COVID-19 severity have also been reported.^18,19^ This study found that patients with rheumatologic disease had lower clinical severity, which is consistent with a previous report^20^ that found that after adjustment for age, diabetes, and kidney impairment, patients with rheumatologic disease on some treatment regimens had a lower risk of hospitalization. Increased risk of intubation and death has been inconsistently found among patients with blood types AB, A, and B relative to type O.^21,22,23^ In contrast, the current study found that blood type AB had a protective association with intubation and death.

The current study also found significant treatment regimen heterogeneity for inpatients with COVID-19. Some medications have fallen out of favor (eg, hydroxychloroquine and azithromycin); others are the subject of ongoing studies (eg, anakinra and tocilizumab). For most treatments, the balance of risks and benefits has not been evaluated rigorously in randomized clinical trials. Ongoing monitoring for adverse effects in observational data such as N3C will be important.

The N3C has unique features that distinguish it from other COVID-19 data resources. First, it harmonizes data from a very large number of clinical sites (86 had signed data transfer agreements as of March 30, 2021), which is important because significant site-level variation in critical metrics, such as invasive ventilatory support and mortality, has been reported.^24,25,26,27^ Central curation ensures that N3C data are robust and quality assured across sites, which is in contrast to the known challenges of relying on site-level CDM quality assurance processes in distributed networks (eg, the Observational Health Data Sciences and Informatics and National Patient-Centered Clinical Research Network). Most US reports^9,26^ of COVID-19 clinical characteristics, disease course, treatments, and outcomes come from a single hospital or health care system in a single geographic region. Another network has reported a large COVID-19 cohort, but the patient-level data are not centralized and thus are less amenable to machine learning.^28^

Developed under the intense time pressure of a health crisis, earlier data aggregation efforts^2,25,29,30,31,32^ may not have been designed to support future research. The N3C Data Enclave^4^ provides transparent, easily shared, versioned, and fully auditable data and analytic provenance. This is an important advantage because a lack of auditable data and analytic provenance has resulted in retraction of high-profile COVID-19 publications.^33,34^

Finally, this study also developed accurate ML models to predict clinical severity based only on information available on the first calendar day of admission. The most powerful predictors in these models are patient age and widely available vital sign and laboratory values. These models, although intended as examples of how N3C can be used, could also be the basis for generalizable clinical decision support tools. However, development of such tools would require additional work at deploying health care systems, including user engagement, workflow analysis, variable mapping and internal validation, and consideration of any desired visualizations and alerts.^35^

### Limitations

This study has limitations. Because the data are aggregated from many health care systems and 4 CDMs that vary in granularity, some sites have systematic missingness of some variables (eMethods in Supplement 1). Detailed respiratory support information, such as oxygen flow, fraction of inspired oxygen, and ventilator settings (typically recorded in electronic health record flowsheets), is not fully available. Orders related to limitations in care, such as do not attempt resuscitation, are not yet present in the N3C. Some inpatient mortality in the study is likely attributable to patients who had do not attempt resuscitation orders in place. Exclusion of those patients might improve severity model prediction. Finally, the exact time at which laboratory values were measured is inconsistently provided by sites, so laboratory test results are standardized to calendar day but not time of day. Hour-level resolution of the association between a laboratory test result and maximum clinical severity is not currently possible.

## Conclusions

The N3C is a nationally representative, transparent, reproducible, harmonized data resource that enables effective and efficient collaborative observational COVID-19 research. This study found that COVID-19 mortality decreased over time during 2020 and that patient demographic characteristics and comorbidities were associated with higher clinical severity. The model developed in this study may be a clinically useful, machine learning–based predictor of SARS-CoV-2 severity.

## Notes

Supplement 1. **eMethods.** Supplementary Methods **eTable 1.** N3C Overall Cohort Characteristics **eTable 2.** Input Variables for Machine Learning **eTable 3.** N3C Cohort and Variables Supported by Source Data Models **eTable 4.** Multivariable Logistic Regression Models for Poor Outcome **eTable 5.** Medications and Organ System Support for Hospitalized Patients, by Severity Group **eTable 6.** Machine Learning Model Performance Metrics **eFigure 1.** Cohort Construction **eFigure 2.** Age, Sex, Race, and Ethnicity Distributions of the Overall N3C Cohort **eFigure 3.** Trajectories of Additional Laboratory Tests During a Hospital Encounter **eFigure 4.** Heatmaps Showing Nadir, Average, and Peak Values of Vital Signs, Body Size Metrics, and Laboratory Test Values, by Severity Group **eFigure 5.** Relatively Few Patients Have Harmonized Blood Type **eFigure 6.** Antimicrobial Treatments and Immunomodulatory Treatments in Hospitalized Patients **eFigure 7.** Area Under the Receiver Operator Characteristic (AUROC) Curves for First-Day Machine Learning Models to Predict Subsequent Clinical Severity **eFigure 8.** Variable Importance in the Machine Learning Models Predicting Clinical Severity

Supplement 2. The National COVID Cohort Collaborative (N3C) Consortium Members
