# Supplementary material for: Clinical Characterization and Prediction of Clinical Severity of SARS-CoV-2 Infection Among US Adults Using Data From the US National COVID Cohort Collaborative
Source: JAMA Netw Open. 2021 Jul 13;4(7):e2116901. doi: 10.1001/jamanetworkopen.2021.16901 (PMC8278272; doi:10.1001/jamanetworkopen.2021.16901)
Supplement: Supplement 1. — eMethods. Supplementary Methods eTable 1. N3C Overall Cohort Characteristics eTable 2. Input Variables for Machine Learning eTable 3. N3C Cohort and Variables Supported by Source Data Models eTable 4. Multivariable Logistic Regression Models for Poor Outcome eTable 5. Medications and Organ System Support for Hospitalized Patients, by Severity Group eTable 6. Machine Learning Model Performance Metrics eFigure 1. Cohort Construction eFigure 2. Age, Sex, Race, and Ethnicity Distributions of the Overall N3C Cohort eFigure 3. Trajectories of Additional Laboratory Tests During a Hospital Encounter eFigure 4. Heatmaps Showing Nadir, Average, and Peak Values of Vital Signs, Body Size Metrics, and Laboratory Test Values, by Severity Group eFigure 5. Relatively Few Patients Have Harmonized Blood Type eFigure 6. Antimicrobial Treatments and Immunomodulatory Treatments in Hospitalized Patients eFigure 7. Area Under the Receiver Operator Characteristic (AUROC) Curves for First-Day Machine Learning Models to Predict Subsequent Clinical Severity eFigure 8. Variable Importance in the Machine Learning Models Predicting Clinical Severity [file jamanetwopen-e2116901-s001.pdf]

Supplementary Online Content

Bennett TD, Moffitt RA, Hajagos JG, et al; National COVID Cohort Collaborative (N3C) Consortium. Clinical characterization and prediction of clinical severity of SARS-CoV-2 infection among US adults using data from the US National COVID Cohort Collaborative. *JAMA Netw Open*. 2021;4(7):e2116901. doi:10.1001/jamanetworkopen.2021.16901

eMethods. Supplementary Methods

eTable 1. N3C Overall Cohort Characteristics

eTable 2. Input Variables for Machine Learning

eTable 3. N3C Cohort and Variables Supported by Source Data Models

eTable 4. Multivariable Logistic Regression Models for Poor Outcome

eTable 5. Medications and Organ System Support for Hospitalized Patients, by Severity Group

eTable 6. Machine Learning Model Performance Metrics

eFigure 1. Cohort Construction

eFigure 2. Age, Sex, Race, and Ethnicity Distributions of the Overall N3C Cohort

eFigure 3. Trajectories of Additional Laboratory Tests During a Hospital Encounter

eFigure 4. Heatmaps Showing Nadir, Average, and Peak Values of Vital Signs, Body Size Metrics, and Laboratory Test Values, by Severity Group

eFigure 5. Relatively Few Patients Have Harmonized Blood Type

eFigure 6. Antimicrobial Treatments and Immunomodulatory Treatments in Hospitalized Patients

eFigure 7. Area Under the Receiver Operator Characteristic (AUROC) Curves for First-Day Machine Learning Models to Predict Subsequent Clinical Severity

eFigure 8. Variable Importance in the Machine Learning Models Predicting Clinical Severity

This supplementary material has been provided by the authors to give readers additional information about their work.

© 2021 Bennett TD et al. *JAMA Network Open.*

eMethods. Supplemental Methods

N3C Architecture, Data Integration and Harmonization Pipeline

The N3C is hosted in a cloud-based, FedRAMP Moderate secure enclave[1] managed by the National Center for Advancing Translational Sciences (NCATS). The N3C Enclave contains l1 Foundry, a data science platform that enables complex and reproducible analysis using a variety of open-source languages (e.g. Python[2], R[3], SQL[4], Java[5], and also point-and-click and dashboard-style tools. Foundry uses Apache Spark[6] to support distributed operations on very large data sources.

Contributing sites submit a Health Insurance Portability and Accountability Act (HIPAA)-defined limited data set in one of four common data models (CDMs: PCORNet[7] , the Observational Medical Outcomes Partnership [OMOP][8], ACT/i2b2[9], or TriNetX[10]. Sites send updated data payloads approximately weekly. N3C harmonizes site data into OMOP version 5.3.1 in partnership with subject matter experts from each CDM community. In this process, all data (e.g. laboratory measurements, clinical observations such as vital signs, medications, and clinical conditions, are harmonized and mapped to the OMOP vocabulary[8]. Site data that pass a robust data quality assessment pipeline are integrated into the "release" set for use by the community. Details about the data transfer, harmonization, quality, and integration processes have been reported[11]. Decisions about which patients become part of the N3C cohort are made by each data-providing site, following the N3C-provided phenotype definitions. In November 2020, N3C released Phenotype definition 3.0, which specifically defines "controls" as patients who have at least one negative COVID test (PCR, antigen, or antibody), and do not also have a positive test or a diagnosis code of U07.1. Phenotype 1.0 and 2.0, which were used from April 2020 through November 2020, did not define "controls," but did enable selection of COVID negative patients as part of its criteria. This group of COVID-negative patients was part of the cohort in the N3C enclave during the completion of this analysis. At the time of this manuscript, a mix of phenotype versions 1.0 and 2.0 were being used by data providers.

Identifying Hospital Encounters, Comorbidities, Medications, Mechanical Ventilation, Vital Signs, and Laboratory Tests

© 2021 Bennett TD et al. *JAMA Network Open.*

We defined a single index encounter for each laboratory-confirmed positive patient by selecting encounters that start up to 30 days before or 7 days after the positive test result, or a positive test result occurs during the visit. When multiple encounters met these criteria, we broke ties by preferentially selecting the encounter in which the most severe outcome was observed, then the longest visit, and finally the most recent visit.

We reconstructed hospital encounters from component "visits" (e.g. a radiology study and a surgical procedure recorded as separate visits) using an algorithm that will be made available to all N3C users. We built hospital encounters from recorded OMOP visits by first filtering to Inpatient (9201), Inpatient Hospital (8717), Intensive Care (32037), Emergency Room (ER) and Inpatient (262), or Inpatient Critical Care Facility (581379) visits of any duration, ER visits (9203) spanning at least 2 calendar days, or Outpatient Visits (9202) spanning exactly two calendar days. These visits were then merged such that any visits with overlapping calendar days would end up in the same hospital stay. Finally, all merged visits that did not contain at least one inpatient or ER visit were unmerged. This process results in combined hospital stays that are separated by a period of at least one calendar day. Finally, visits of any type that occur during a combined hospital stay are added to the hospital stay.

All OMOP concept sets developed for this manuscript are freely available on the platform, versioned, and include attributed input from both informatics and clinical subject matter experts. None of the 4 CDMs support admission, discharge, and transfer (ADT) tables, which complicates analyses of hospital encounters.

We defined comorbidities based on the updated[12] Charlson Comorbidity Index as implemented in the 'icd' R package[13]. Unless otherwise noted, we identified medications using the WHO anatomical therapeutic chemical (ATC) definitions.[14] We built an invasive ventilation concept set from standardized terminology codes (International Classification of Diseases [ICD] and Systematized Nomenclature of Medicine [SNOMED]) included in the OMOP CDM. Among hospitalized patients, we assessed serial measurements of heart rate (HR), respiratory rate (RR), temperature, systolic and diastolic blood pressure (SBP and DBP, respectively), pulse oximetry (SpO_2), and a variety of laboratory tests.

© 2021 Bennett TD et al. *JAMA Network Open.*

Statistical eMethods

Software

We used reproducible pipelines in SQL, R, and Python to conduct all analyses. Our pipelines relied on the *SparkR*[15] and *pyspark*[16] interfaces to Apache Spark[6]. We built machine learning models using Python's 'scikit-learn'[17] and XGBoost packages and visualizations using R's 'ggplot2'[18], 'ggalluvial'[19], and 'ggnewscale' packages[20] and Python's Matplotlib package.[21]

Machine *Learning eMethods*

Categorical variables were converted to k-1 dummy variables using Pandas' get_dummies (one-hot encoding). For logistic regression and support vector machines, numeric variables were centered to mean zero with unit variance using scikit-learn's StandardScaler. Optimal model specific hyperparameters were selected with a grid search performed using scikit-learn's GridSearchCV using 5-fold cross validation on the training set with AUROC as the scoring metric. Each grid search included multiple iterations with categorical settings such as solver and with first coarse settings for numeric parameters following a logarithmic scale followed by more specific settings around the values found to perform best. In final training, 5-fold cross validation was performed on the training set to estimate AUROC performance range, see eTable 6 legend and table for more model metrics including mean AUROC and standard deviation of AUROC from the 5-fold cross validation. Scikit-learn provides a common API to extract feature importance for a model. Each ML method has an algorithm for determining feature importance. For XGBoost we used the type "gain", the average gain across all splits the feature is used in; for RandomForest we used Gini; for logistic regression methods (no penalty, L1, L2) we reported ordered absolute value of coefficients (all input data had the mean set to 0 and were scaled to unit variance.) When ranking features from L1-regularized models with a coefficient of 0, we show these in eFigure 8 with an equal lack of importance as having the same ranking in the table.

© 2021 Bennett TD et al. *JAMA Network Open.*

Ethics and Regulatory

The N3C Data Enclave is approved under the authority of the NIH Institutional Review Board for Protocol 000082 associated with NIH iRIS reference number: 546652 entitled: "NCATS National COVID-19 Cohort Collaborative (N3C) Data Enclave Repository." Further information can be found at https://ncats.nih.gov/n3c/resources. Each N3C site maintains an IRB-approved data transfer agreement (Johns Hopkins University Reliance Protocol # IRB00249128 or individual site agreements with NIH). The analyses reported in this manuscript were approved separately by the institutional IRB of each investigator with data access (see below). This approval includes a waiver of informed consent.

Use of the N3C data for this study is authorized under the following IRB Protocols:

Site	IRB name	Exempted vs. approved	Protocol number
University of Alabama-Birmingham	The University of Alabama at Birmingham Office of the Institutional Review Board for Human Use	exempted	IRB-300006285
University of Colorado	Colorado Multiple Institutional Review Board	approved	20-2225
Johns Hopkins University	Johns Hopkins Office of Human Subjects Research - Institutional Review Board	approved	IRB00249128
University of Kentucky	Medical Institutional Review Board of the University of Kentucky	exempted	62294
University of Michigan	University of Michigan Medical School Institutional Review Board	approved	HUM00188854
University of North Carolina	University of North Carolina Chapel Hill Institutional Review Board	exempted	20-3106
Oregon State University	Oregon State University Institutional Review Board	approved	IRB-2020-0830
University of Rochester	University of Rochester Research Subjects Review Board	exempted	STUDY00005366
Stony Brook University	Office of Research Compliance, Division of Human Subject Protections, Stony Brook University	exempted	IRB2020-00604
University of Texas-Medical Branch	Institutional Review Board of the University of Texas Medical Branch	exempted	20-0245

© 2021 Bennett TD et al. *JAMA Network Open.*

Other referenced resources are available at:

COVID-19 Map - Johns Hopkins Coronavirus Resource Center https://coronavirus.jhu.edu/map.html

Institutional Development Award Program Infrastructure for Clinical and Translational Research (IDeA-CTR)

https://www.nigms.nih.gov/Research/DRCB/IDeA/Pages/IDeA-CTR.aspx

xgboost https://github.com/dmlc/xgboost

eTables

Variable, n(%) unless otherwise indicated	All	Lab-confirmed Positive	Lab-confirmed Negative	Suspected Positive	No Test for SARS- CoV-2
**Age, mean (SD)**	43.2 (22.9) n=1,925,699	41.4 (20.4) n=199,935	44.2 (22.6) n=1,339,933	39.2 (26.2) n=174,831	41.6 (23.1) n=211,000
**Sex**					
**Female**	1,074,141	106,316	750,606	96,073	121,146
**Male**	851,007	93,607	588,750	78,904	89,746
**Other***	1,378	139	815	205	219
**Race**					
**White**	1,251,401	104,491	898,340	108,992	139,578
**Black or African- American**	301,994	36,243	198,569	35,532	31,650
**Native Hawaiian or Pacific Islander**	3,034	459	2,000	265	310
**Asian**	48,897	4,690	35,106	4,188	4,913
**Other**	20,626	2,363	1,3813	1,405	3045
**Missing/Unknown**	300,574	51,816	192,343	24,800	31,615
**Ethnicity**					
**Hispanic**	156,401	34,657	93,137	16,668	11,939
**Non-Hispanic**	1,498,261	130,297	1,072,760	126,982	168,222
**Missing/Unknown**	300,574	51,816	192,343	24,800	31,615
**Insurance Payer**					
**Medicare**	118,381	7,416	87,102	9,924	13,939
**Commercial**	212,527	17,247	137,233	15,437	42,610
**Medicaid**	106,558	9,532	66,677	12,436	17,913
**Other**	1,783,181	186,593	1,252,174	149,335	195,079

eTable 1: N3C Cohort Characteristics
eTable 1: This table shows demographic characteristics and insurance payer for the overall N3C cohort, stratified by the N3C phenotype groups (publicly available on GitHub[22]). SARS-CoV-2 = severe acute respiratory syndrome associated with coronavirus-2. *Other includes non-binary, no matching concept, and no information. Please note that the lab-confirmed positive and negative counts in this table differ from Table 1 in the main manuscript because Table 1 is restricted to sites with death and ventilation data available.

© 2021 Bennett TD et al. *JAMA Network Open.*

eTable 2: Input Variables for Machine Learning

This table shows the 42 categories of 64 input variables for the machine learning models. The worst value for each variable on the first calendar day of hospital admission was used. We defined the worst value as the lowest value for diastolic blood pressure, hemoglobin, pH, platelet count, SpO2, and systolic blood pressure. For the remainder, we used the highest value. NTproBNP = N-Terminal-prohormone B-type Natriuretic Peptide. *(White, Black or African-American, Native Hawaiian or Pacific Islander, Other, or Missing/Unknown)

Variable (units)	%present	Imputation Strategy
Age at visit start (years)	100.0%	None
Sex (Female, Male, or Other)	100.0%	Missing values filled with 'Other'
White blood cell count (x10E3/uL)	94.2%	Median
Platelet count (x10E3/uL)	94.1%	Median
Hemoglobin (g/dL)	93.2%	Median
Creatinine (mg/dL)	92.9%	Median
Sodium (mmol/L)	92.8%	Median
BUN (mg/dL)	92.7%	Median
Chloride (mmol/L)	92.7%	Median
Potassium (mmol/L)	92.5%	Median
Glucose (mg/dL)	92.1%	Median
Ethnicity (Hispanic, Not Hispanic, or Missing/Unknown)	88.8%	Missing values filled with 'Missing/Unknown'
Aspartate Aminotransferase (AST/SGOT, IU/L)	83.4%	Median
Bilirubin (total, mg/dL)	82.9%	Median
Race*	76.7%	Missing values filled with 'Missing/Unknown'
Alanine Aminotransferase (ALT/SGPT, IU/L)	75.7%	Median
Absolute Lymphocyte count (x10E3/uL)	74.6%	Median
Body Weight (kg)	73.3%	Median
Absolute Neutrophil count (x10E3/uL)	70.2%	Median
Diastolic blood pressure (DBP)	65.9%	Median
Systolic blood pressure (SBP)	65.9%	Median
Albumin (g/dL)	57.5%	Median
Oxygen saturation (SpO2)	53.5%	Median
Ferritin (ng/mL)	49.3%	Male and missing: 150; Female and missing: 75
Respiratory Rate	49.3%	Median
C-reactive protein (CRP, mg/L)	49.1%	Missing values filled with 10
Charlson Cancer	48.6%	FALSE
Charlson Congestive heart failure (CHF)	48.6%	FALSE
Charlson Dementia	48.6%	FALSE
Charlson Diabetes Mellitus	48.6%	FALSE
Charlson Diabetes Mellitus with complications	48.6%	FALSE
Charlson HIV	48.6%	FALSE
Charlson Liver disease (mild)	48.6%	FALSE
Charlson Liver disease (severe)	48.6%	FALSE
Charlson Metastases	48.6%	FALSE
Charlson Myocardial Infarction	48.6%	FALSE
Charlson Hemiplegia or paralysis	48.6%	FALSE
Charlson Peptic ulcer disease	48.6%	FALSE
Charlson Pulmonary disease	48.6%	FALSE
Charlson Peripheral vascular disease	48.6%	FALSE
Charlson Comorbidity Index, Q score	48.6%	Missing values filled with 0
Charlson Renal disease	48.6%	FALSE
Charlson Rheumatologic Disease	48.6%	FALSE
Charlson Stroke	48.6%	FALSE
Body mass index (BMI, kg/m2)	48.3%	Median
Temperature	46.1%	Median
Lactate (mM/L)	45.5%	Missing values filled with 13.5
D-Dimer (mg/L FEU)	43.7%	Median
Troponin all types (ng/mL)	43.2%	Median
Heart rate	34.1%	Median
Bilirubin (conjugated/direct, mg/dL)	27.0%	Median
pH	26.4%	Median
Procalcitonin (ng/mL)	24.9%	Missing values filled with 0.02
Hemoglobin-glycosylated (A1C, %)	20.2%	Median
Erythrocyte Sedimentation Rate (mm/hr)	19.6%	Missing values filled with 19

© 2021 Bennett TD et al. *JAMA Network Open.*

	NTproBNP (pg/mL)	18.3%	Missing values filled with 125
	BNP (pg/mL)	16.3%	Missing values filled with 100

eTable 3. N3C Cohort and Variables Supported by Source Data Models

a: N3C Cohort by Source Data Model

This table shows the representation among N3C sites of each common data model (CDM). CDMs include the National Patient-Centered Clinical Research Network (PCORNet),[7] the Observational Health Data Sciences and Informatics (OHDSI) network,[23] the Accrual to Clinical Trials (ACT) network,[9] and TriNetX[10]. This table includes a total of 36 sites. Two sites are dropped prior to analysis due to missing date data (see eFigure 1).

Data model	# N3C Sites	# Patients represented
**OMOP**	**6**	305,376
**PCORnet**	**12**	1,036,073
**i2b2/ACT**	**6**	359,920
**TriNetX**	**10**	444,690

b: Variables Supported by Source Data Models[1]

S = supported, NS = not supported. **[1]** Variables *supported* by a data model may not be *required* by that model to conform to the model's specification. Thus, some systematic missingness may be at the site level rather than the model level. **[2]** Many of the items marked "not supported" for ACT can technically be stored in the i2b2 data model, which underlies ACT; however, they are not supported by the ACT ontology at this time, and are not harmonized by N3C. **[3]** All models support both quantitative and qualitative lab results; however, many sites only map a subset of their qualitative lab results to the model's vocabulary. *A small set of vitals are defined by the model; additional vital data can optionally be modelled as "observations"

	**OMOP**	**PCORnet**	**ACT[2]**	**TriNetX**
Patient Demographics	S	S	S	S
Visit (encounter) details	S	S	S	S
Discharge disposition	S	S	NS	NS
Diagnoses	S	S	S	S
Medications	S	S	S	S
Laboratory results[3]	S	S	S	S
Procedures	S	S	S	S
Vital signs	S	S*	NS	S
Location of patient residence (ZIP code-level)	S	S	NS	S
Death	S, date required	S, date not required	S, date not required	S, date required
Admission - Discharge - Transfer transactions	NS	NS	NS	NS
Insurance	S	S	NS	NS

© 2021 Bennett TD et al. *JAMA Network Open.*

eTable 4: Multivariable Logistic Regression Models for Poor Outcome

This table shows odds ratios (ORs) and 95% confidence intervals (CIs) for 2 multivariable logistic regression models, one with missing/unknown as a category when relevant and one with complete cases only. LCL = lower confidence limit, lower bound of 95% CI. UCL = upper confidence limit, upper bound of 95% CI. See Results for details. These models were built after the prediction models and are for inference only.

			**Missing encoded**			**Missing cases dropped**	
			**OR (LCL,UCL)**	**p-value**		**OR (LCL,UCL)**	**p-value**
**Age**	per year		1.034 (1.032,1.036)	p < 0.0001		1.032 (1.025,1.038)	p < 0.0001
**Comorbidities**	Diabetes mellitus		1.05 (0.98,0.12)	p = 0.2106		0.85 (0.68,1.06)	p = 0.1557
	Liver disease		1.20 (1.08,1.34)	p = 0.0010		1.07 (0.78,1.45)	p = 0.6915
	Cancer		0.96 (0.87,1.05)	p = 0.3922		0.85 (0.64,1.12)	p = 0.2403
	Pulmonary		0.93 (0.86,1.01)	p = 0.0886		0.91 (0.71,1.15)	p = 0.4259
	Renal		1.06 (0.97,1.15)	p = 0.2053		0.91 (0.71,1.18)	p = 0.4938
	Congestive Heart Failure		1.07 (0.98,1.17)	p = 0.1226		0.88 (0.66,1.15)	p = 0.3431
	Rheumatic Disease		0.83 (0.72,0.96)	p = 0.0151		0.93 (0.62,1.38)	p = 0.7210
	Dementia		1.26 (1.13,1.41)	p < 0.0001		0.80 (0.54,1.19)	p = 0.2761
	none of the above		1.00	ref.		1.00	ref.
**Gender**	Male		1.60 (1.507,1.69)	p < 0.0001		1.70 (1.40,2.01)	p < 0.0001
	Female		1.00	ref.		1.00	ref.
**Ethnicity**	Hispanic or Latino		1.04 (0.94,1.15)	p = 0.4663		1.04 (0.72,1.49)	p = 0.8381
	Not Hispanic or Latino		1.00	ref.		1.00	ref.
	unknown		1.14 (1.04,1.25)	p = 0.0057			
**Race**	Black or African-American		1.12 (1.05,1.20)	p = 0.0011		1.21 (0.97,1.51)	p = 0.0930
	Asian		1.33 (1.12,1.57)	p = 0.0011		2.36 (1.38,4.04)	p = 0.0017
	Other		1.25 (0.999, 1.56)	p = 0.0477		1.22 (0.74,1.97)	p = 0.4255
	White		1.00	ref.		1.00	ref.
	unknown		1.19 (1.08,1.31)	p = 0.0005			
**BMI**	over 30		1.36 (1.27,1.46)	p < 0.0001		1.41 (1.16,1.73)	p = 0.0008
	30 or under		1.00	ref.		1.00	ref.
	unknown		1.23 (1.14,1.32)	p < 0.0001			
**Blood type**	A		0.90 (0.76,1.08)	p = 0.2660		0.93 (0.75,1.15)	p = 0.4910
	B		0.97 (0.76,1.23)	p = 0.7884		1.12 (0.84,1.49)	p = 0.4405
	AB		0.55 (0.32,0.92)	p = 0.0256		0.53 (0.29,0.94)	p = 0.0353
	O		1.00	ref.		1.00	ref.

© 2021 Bennett TD et al. *JAMA Network Open.*

	unknown		0.37 (0.32,0.41)	p < 0.0001			
**Rh factor**	negative		0.94 (0.70,1.25)	p = 0.6621		1.11 (0.78,0.94)	p = 0.5477
	positive		1.00	ref.		1.00	ref.

eTable 5: Antimicrobials and Immunomodulation, Respiratory, Cardiovascular, and Renal Organ System Support for Hospitalized Patients, by Severity Group

eTable 5a: Antimicrobials and Immunomodulation

This table shows the percent of patients in each category who received each medication type. ED = Emergency Department. WHO = World Health Organization. ECMO = extracorporeal membrane oxygenation. LOS = length of stay. We stratified patients using the Clinical Progression Scale (CPS) established by the World Health Organization (WHO) for COVID-19 clinical research.[24] Severity assigned by patient-specific encounter maximum severity.

eTable 5b: Respiratory, Cardiovascular, and Renal Organ System Support for Hospitalized Patients, by Severity Group

This table shows the percent of patients in each category who received each treatment type. ED = Emergency Department. WHO = World Health Organization. ECMO = extracorporeal membrane oxygenation. LOS = length of stay. We stratified patients using the Clinical Progression Scale (CPS) established by the World Health Organization (WHO) for COVID-19

© 2021 Bennett TD et al. *JAMA Network Open.*

clinical research.[24] Severity assigned by patient-specific encounter maximum severity. CRRT = Continuous Renal Replacement Therapy. HD = hemodialysis.

A					B			
	**Moderate Hospitalized without invasive ventilation WHO Severity 4-6**	**Severe Hospitalized with invasive ventilation or ECMO WHO Severity 7-9**	**Hospital Mortality or Discharge to Hospice WHO Severity 10**			**Moderate Hospitalized without invasive ventilation WHO Severity 4-6**	**Severe Hospitalized with invasive ventilation or ECMO WHO Severity 7-9**	**Hospital Mortality or Discharge to Hospice WHO Severity 10**
**Antimicrobials**					**Respiratory Support**			
Remdesivir	15.31%	25.13%	20.08%		Inhaled Nitric Oxide	0.00%	0.04%	0.08%
Lopinavir/Ritonavir	0.36%	1.94%	1.09%		Epoprostenol	0.11%	7.06%	6.52%
Hydroxychloroquine	6.85%	21.76%	14.65%					
Chloroquine	0.04%	0.61%	0.19%		**Cardiovascular Support**			
Any Antibacterial					Amiodarone	0.63%	8.60%	12.64%
Any Antiviral	3.18%	7.96%	5.78%		Dopamine	2.07%	7.42%	7.02%
Any Antifungal	2.20%	15.99%	13.85%		Dobutamine	0.17%	3.48%	2.86%
					Epinephrine	1.02%	10.00%	14.38%
**Immunomodulation**					Esmolol	0.30%	2.69%	1.72%
Dexamethasone	9.45%	13.66%	9.43%		Isoproterenol	0.02%	0.07%	0.08%
Prednisone	7.76%	16.42%	10.15%		Milrinone	0.04%	1.11%	0.61%
Methylprednisolone	4.42%	21.15%	16.00%		Norepinephrine	0.44%	14.66%	11.89%
Hydrocortisone	1.00%	14.34%	18.31%		Phenylephrine	1.04%	12.87%	10.20%
Any systemic steroid	35.83%	67.46%	56.21%		Vasopressin	1.04%	12.87%	10.20%
Anakinra	0.05%	0.04%	0.13%		ECMO	0.00%	5.02%	2.49%
Tocilizumab	0.78%	13.55%	6.33%		CRRT or HD	1.53%	9.75%	9.59%

© 2021 Bennett TD et al. *JAMA Network Open.*

eTable 6: Machine Learning Model Performance Metrics

This table shows performance metrics for each machine learning model type over Inpatient stays ending between January 2020 and November 2020. Mar-May = March to May 2020. Jun-Oct = June to October 2020. AUROC = area under the receiver operator characteristic curve.

		Random Forest	XGBoost	Support Vector Machines	Logistic Regression		
					None	L1	L2
Balanced Accuracy	All	67.90%	70.90%	62.40%	65.80%	65.20%	65.90%
	Jun-Oct	64.80%	68.20%	60.30%	63.20%	62.90%	63.60%
	Mar-May	70.40%	73.20%	64.10%	68.00%	67.20%	67.90%
F1	All	51.70%	56.50%	39.70%	47.00%	45.80%	47.30%
	Jun-Oct	43.80%	49.50%	33.80%	40.00%	39.40%	40.90%
	Mar-May	57.30%	61.90%	44.30%	52.40%	50.90%	52.20%
Positive Predictive Value/ Precision	All	78.70%	73.00%	79.80%	70.00%	70.50%	69.60%
	Jun-Oct	69.00%	63.50%	71.00%	60.30%	61.70%	60.80%
	Mar-May	86.20%	80.40%	85.90%	76.30%	76.40%	75.10%
AUROC	All	87.00%	87.40%	83.20%	83.80%	83.80%	83.80%
	Jun-Oct	86.40%	86.10%	81.90%	83.30%	83.20%	83.40%
	Mar-May	87.90%	89.20%	85.10%	85.10%	85.20%	85.00%
Recall/ Sensitivity	All	38.50%	46.10%	26.50%	35.40%	33.90%	35.80%
	Jun-Oct	32.10%	40.60%	22.20%	29.90%	28.90%	30.80%
	Mar-May	42.90%	50.30%	29.80%	39.90%	38.20%	40.00%

© 2021 Bennett TD et al. *JAMA Network Open.*

eFigures

eFigure 1: Cohort Construction

This Sankey plot shows how the cohort accumulated from the N3C sites. The left vertical axis shows the number of patients (M = 1,000,000). Each site has a color. The width of the arrows corresponds to the number of patients from that site. Two sites did not submit sufficient date information for us to calculate the N3C computable phenotypes (publicly available on GitHub[22]), excluded as noted "FALSE" in the middle column. We then excluded a) sites who did not submit sufficient death and ventilation information and b) children < 18 years old ("removed from study"), and c) sites whose patients were overwhelmingly children. We show laboratory-confirmed positive as "PCR pos" in this plot due to limited space, but <5% of the patients at one site were included with SARS-CoV-2 antigen positivity. The remainder had positive SARS-CoV-2 polymerase chain reaction (PCR) tests. See Methods for details.

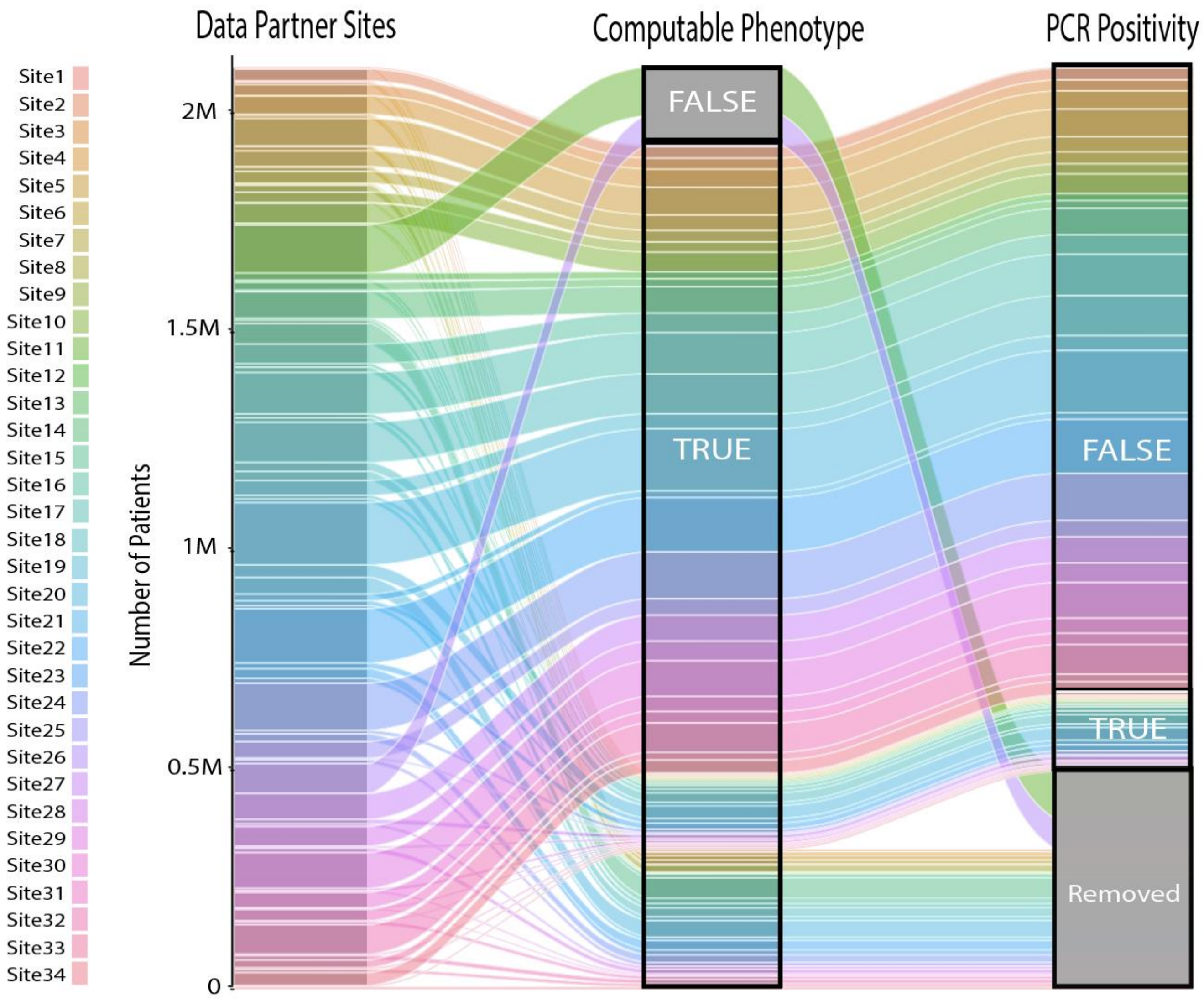

© 2021 Bennett TD et al. *JAMA Network Open.*

eFigure 2: Age, Sex, Race, and Ethnicity Distributions of the overall N3C Cohort

This figure shows the age, sex, race, and ethnicity distributions of the overall N3C cohort, stratified by the N3C phenotype groups (publicly available on GitHub[c]). Racial and ethnic minorities are well-represented. COVID = coronavirus disease.

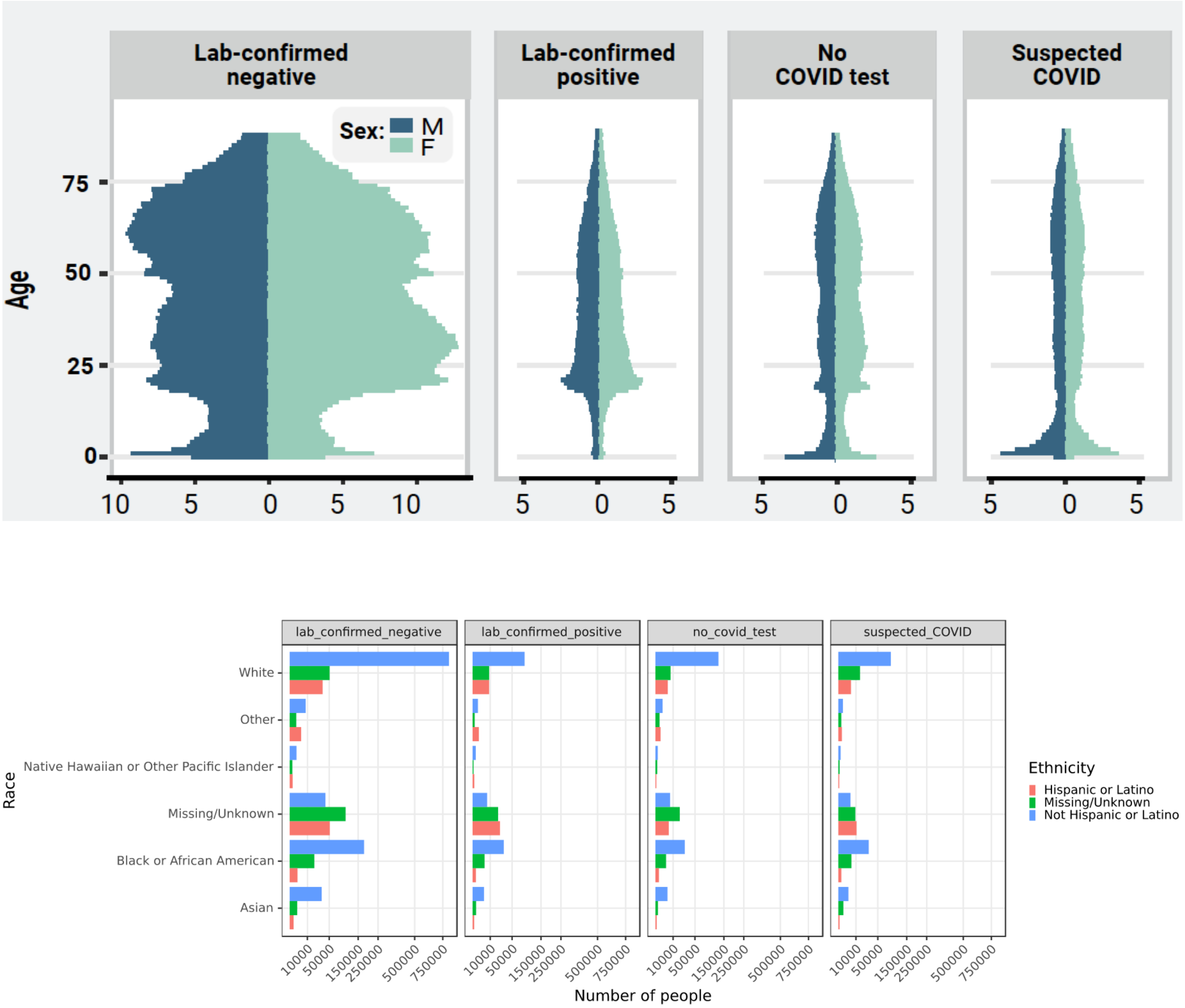

© 2021 Bennett TD et al. *JAMA Network Open.*

eFigure 3: Trajectories of Additional Laboratory Tests During a Hospital Encounter

This figure shows the median (line) and interquartile range (bars) of each laboratory test on each hospital day, stratified by patient maximum severity (hospital mortality or discharge to hospice [black], invasive ventilation or extracorporeal membrane oxygenation [red], hospitalized without any of those [yellow], or emergency department visit only [green], see Table 1). ALT = alanine aminotransferase. AST = aspartate aminotransferase. BUN = blood urea nitrogen. Sed. = sedimentation (erythrocyte sedimentation rate). IL-6 = interleukin-6. NTproBNP = N-Terminal-prohormone B-type Natriuretic Peptide. We tested trajectory differences between severity groups using one-way ANOVA at day 7, see manuscript text.

© 2021 Bennett TD et al. *JAMA Network Open.*

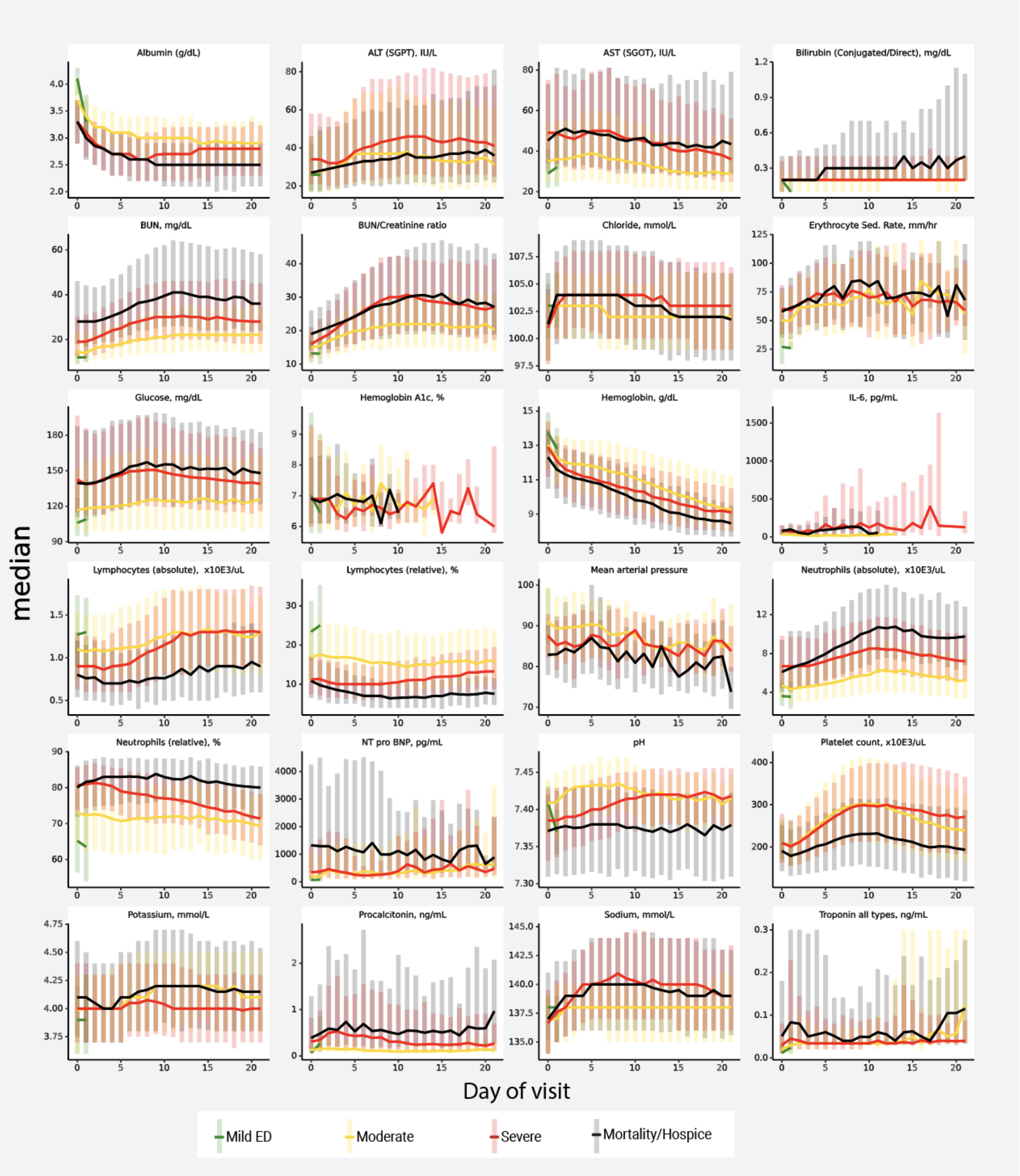

eFigure 4. Heatmaps showing Nadir, Average, and Peak Values of Vital Signs, Body Size Metrics, and Laboratory Test Values, by Severity Group

© 2021 Bennett TD et al. *JAMA Network Open.*

a: Heatmap showing Nadir, Average, and Peak Values of Vital signs and Body Size Metrics, by Severity Group

Values shown for each vital sign and body size metric for each severity group are multiples of the interquartile range (IQR) away from the median value. Circle diameter corresponds to the number of IQRs away from the median, with blue representing below the median and red representing above the median.

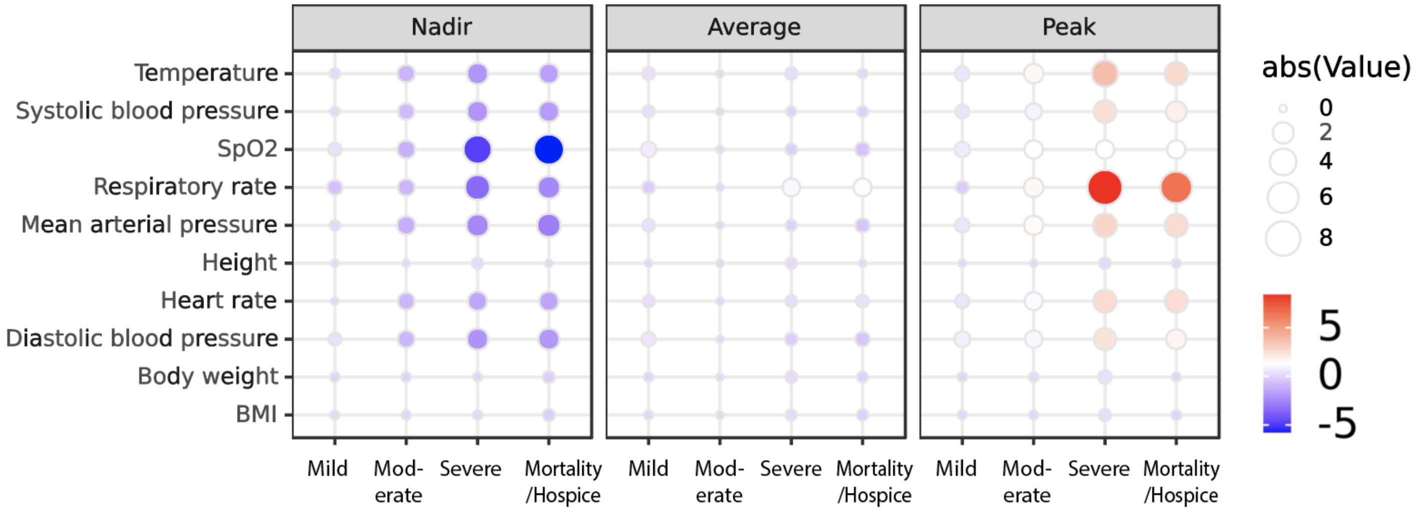

© 2021 Bennett TD et al. *JAMA Network Open.*

b: Heatmap showing Nadir, Average, and Peak Values of Laboratory Test Values, by Severity Group

Values shown for each laboratory test for each severity group are multiples of the interquartile range (IQR) away from the median value. Circle diameter corresponds to the number of IQRs away from the median, with blue representing below the median and red representing above the median. ALT = alanine aminotransferase. AST = aspartate aminotransferase. BUN = blood urea nitrogen. Sed. = sedimentation (erythrocyte sedimentation rate). IL-6 = interleukin-6. NTproBNP = N-Terminal-prohormone B-type Natriuretic Peptide.

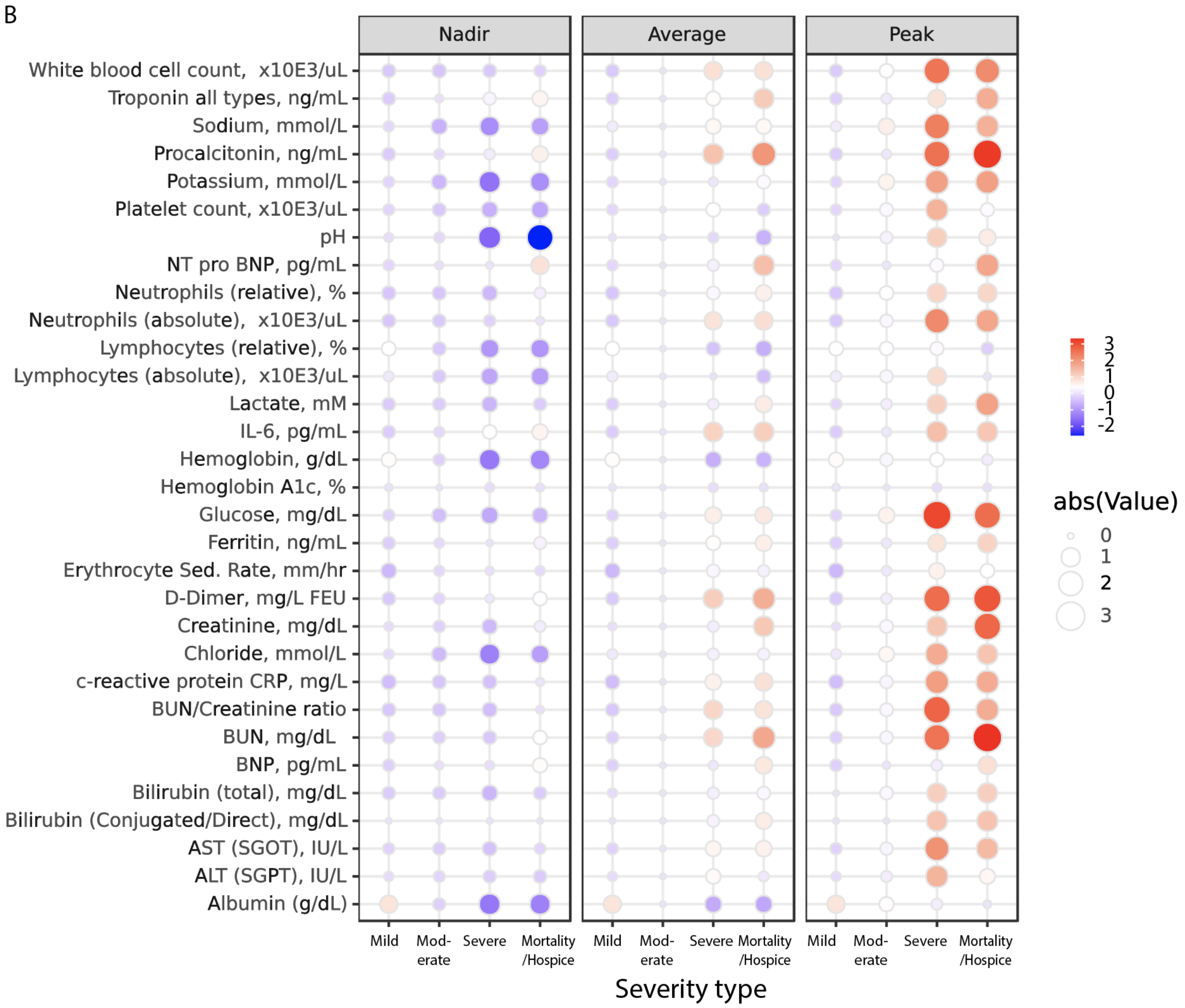

© 2021 Bennett TD et al. *JAMA Network Open.*

eFigure 5: Relatively Few Patients have Harmonized Blood Type

This sunburst plot[25] is read from inside out. Each arc length corresponds to the proportion of that circle represented by that category. Composite patterns are shown as adjacent segments (inside to out), e.g. known blood type, type A, and positive Rh antigen. ED = Emergency Department. Neg = Rh negative. Pos = Rh positive. Stratification is by severity group.

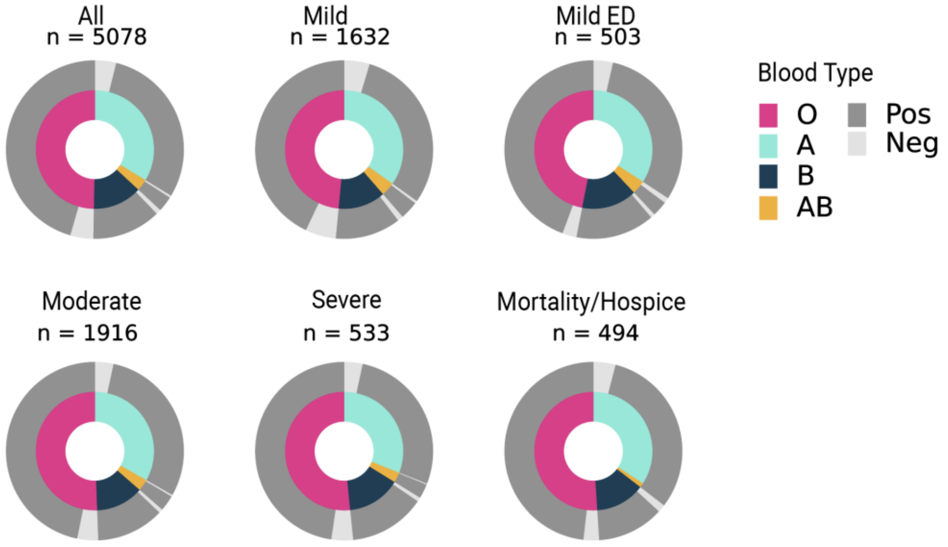

© 2021 Bennett TD et al. *JAMA Network Open.*

eFigure 6. Antimicrobial Treatments and Immunomodulatory Treatments in Hospitalized Patients

A: This sunburst plot[25] is read from inside to outside. Each arc length corresponds to the proportion of that circle represented by that category. Composite treatment regimens are shown as adjacent segments (inside to out, e.g. systemic antibiotic yes, azithromycin yes, hydroxychloroquine yes). Stratification is by severity group.

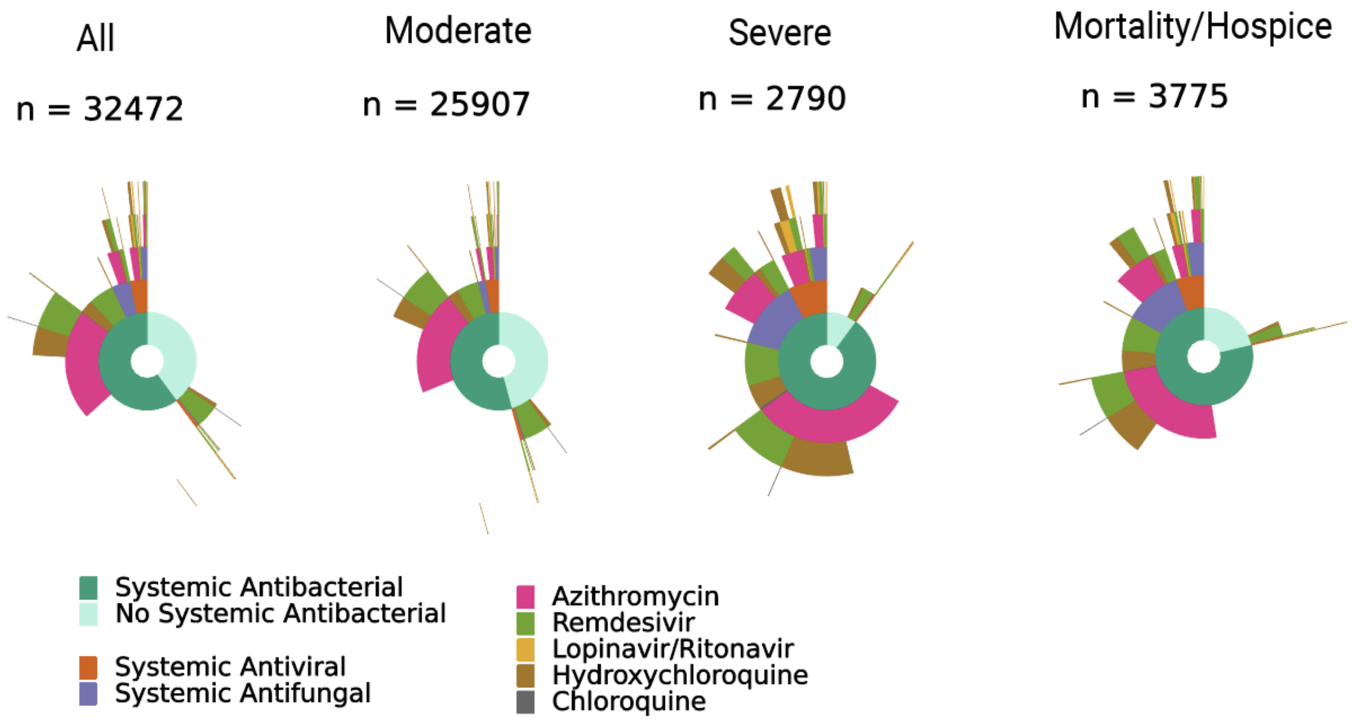

B: This sunburst plot is read from inside to outside.[25] Each arc length corresponds to the proportion of that circle represented by that category. Composite treatment regimens are shown as adjacent segments (inside to out, e.g. systemic corticosteroid yes, dexamethasone yes, anakinra yes). Stratification is by severity group.

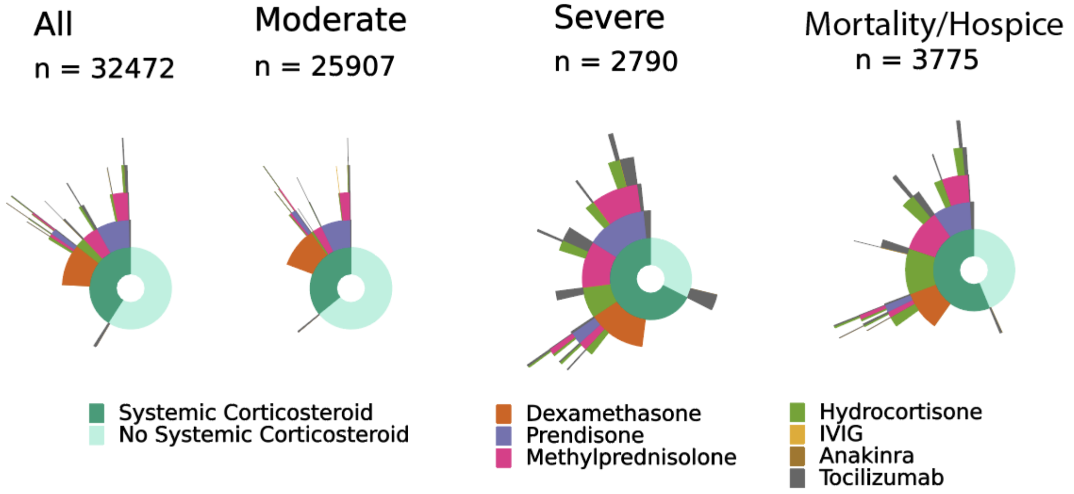

© 2021 Bennett TD et al. *JAMA Network Open.*

eFigure 7 Title: Area Under the Receiver Operator Characteristic (AUROC) Curves for First-Day Machine Learning Models to Predict Subsequent Clinical Severity

eFigure 7 Legend: AUC = AUROC. SVM = support vector machines. Logistic regression is shown with no penalization and L1 and L2 penalization. See Methods for details.

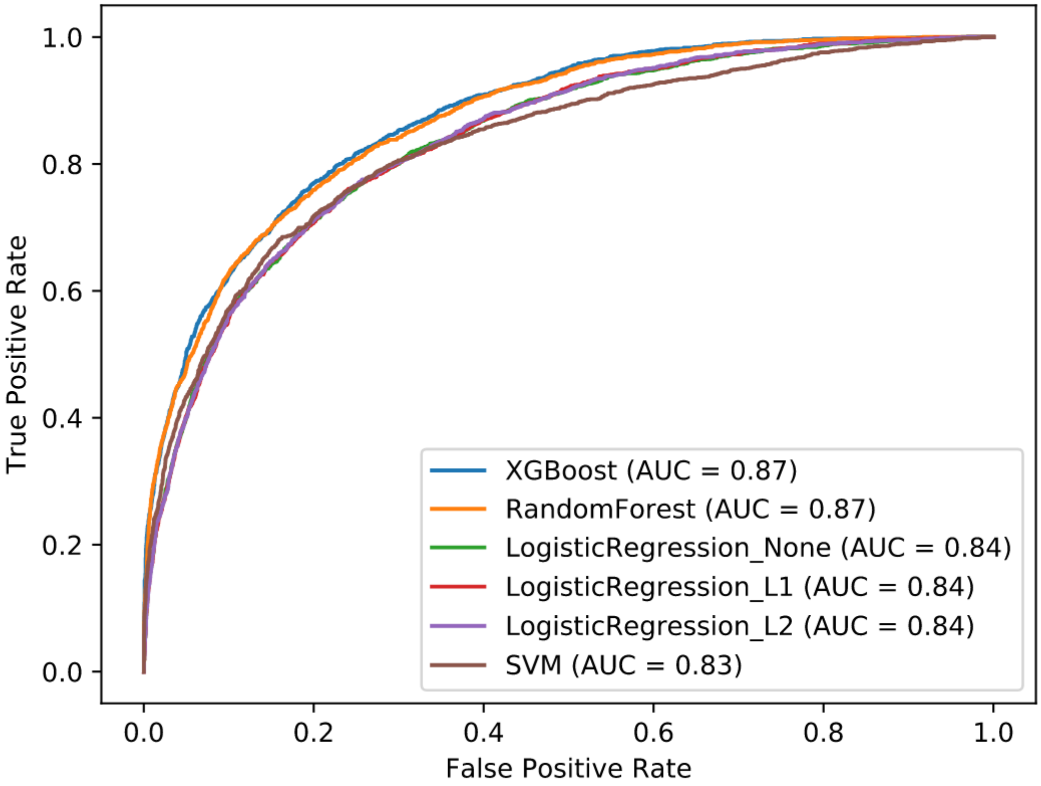

eFigure 8. Variable Importance in the Machine Learning Models Predicting Clinical Severity

The 64 machine learning (ML) model input variables are listed by their mean variable importance rank across ML model types. Each column is a ML model type. Logistic regression is shown without penalization and with L1 and L2 penalties. The table cells show a heat map with darkest (blue) representing highest variable importance and lightest (teal) representing lower variable importance. See Methods and Supplemental Methods for details about variable definitions, model construction, and testing. NTproBNP = N-Terminal-prohormone B-type Natriuretic Peptide.

© 2021 Bennett TD et al. *JAMA Network Open.*

Variable	Random Forest	XG Boost	Logistic Regression: None	Logistic Regression: L1	Logistic Regression: L2	Mean
pH	0	0	1	1	1	0.6
Age at visit start (years)	3	4	0	0	0	1.4
Respiratory rate	5	3	2	2	2	2.8
Oxygen saturation (SpO2)	2	2	6	5	3	3.6
Blood urea nitrogen (BUN)	1	1	13	11	9	7
Systolic blood pressure (SBP)	9	12	5	4	5	7
Aspartate aminotransferase (AST)	6	6	3	10	11	7.2
Albumin	21	7	9	6	6	9.8
Ethnicity = missing or unknown	43	5	4	3	4	11.8
C-reactive protein (CRP)	16	14	14	9	10	12.6
Lactate	20	18	11	7	8	12.8
Absolute neutrophil count	11	27	12	8	7	13
Glucose	4	23	21	16	15	15.8
Platelet count	10	28	16	13	14	16.2
Sodium	13	17	17	19	16	16.4
Hemoglobin	12	31	18	15	18	18.8
Diastolic blood pressure (DBP)	17	11	28	20	19	19
Troponin	15	9	29	23	21	19.4
B-type natriuretic peptide (BNP)	32	21	19	14	13	19.8
Sex = male	40	38	15	12	12	23.4
Body weight	19	41	23	18	20	24.2
Charlson Dementia	37	8	31	24	23	24.6
Temperature	26	32	24	21	22	25
Erythrocyte sedimentation rate (ESR)	36	40	20	17	17	26
D-dimer	25	34	33	25	25	28.4
Ferritin	23	36	35	28	26	29.6
Creatinine	8	53	27	34	27	29.8
Bilirubin conjugated	35	25	32	27	31	30
Absolute lymphocyte count	14	20	43	41	35	30.6
Bilirubin total	28	49	36	22	24	31.8
Body mass index	27	46	34	26	30	32.6
Charlson Diabetes mellitus	41	42	30	29	28	34
Potassium	22	54	38	30	29	34.6
Hemoglobin - glycosylated (A1C)	33	45	40	35	32	37
White blood cell count	7	10	63	49	62	38.2
Charlson Congestive heart failure	45	37	37	38	34	38.2
Charlson Myocardial Infarction	51	30	41	36	33	38.2
Race = missing or unknown	47	51	7	49	39	38.6
Alamine aminotransferase (ALT)	24	44	22	49	55	38.8
Charlson Metastases	56	13	48	32	45	38.8
Charlson Renal Disease	44	26	44	40	41	39
Ethnicity = not Hispanic or Latino	39	29	26	49	54	39.4
Procalcitonin	29	19	55	49	48	40
Heart rate	30	35	50	42	44	40.2
Race = white	38	52	8	49	57	40.8
Charlson Q Score	31	22	54	49	50	41.2
Race = Black or African-American	42	57	10	49	49	41.4
Chloride	18	47	39	49	59	42.4
Race = Asian	54	43	49	33	36	43
Charlson Liver Disease (severe)	59	24	51	37	46	43.4
NTproBNP	34	33	53	48	52	44
Charlson Cancer	52	58	42	31	37	44
Charlson Stroke	49	56	47	43	40	47
Race = other	61	61	25	46	43	47.2
Charlson Diabetes mellitus with complications	50	16	60	49	63	47.6
Charlson hemiplegia or paralysis	58	15	62	49	61	49
Charlson Peripheral vascular disease	46	39	56	49	56	49.2
Charlson HIV	62	60	45	44	38	49.8
Sex = other	63	61	46	39	42	50.2
Charlson Liver Disease (mild)	53	48	52	47	53	50.6
Charlson Rheumatologic disease	55	55	57	49	51	53.4
Charlson Peptic Ulcer Disease	57	50	59	49	58	54.6
Charlson Pulmonary disease	48	59	58	49	60	54.8
Race = Native Hawaiian or Pacific Islander	60	61	61	45	47	54.8

Feature importance rank

Rank	
0	More important
10	
20	
30	
40	
50	
60	
63	Less Important

© 2021 Bennett TD et al. *JAMA Network Open.*

SUPPLEMENTAL REFERENCES

1 Taylor L. FedRAMP: History and Future Direction. *IEEE Cloud Computing* 2014; **1**: 10–4.

2 Welcome to Python.org. https://www.python.org/ (accessed Nov 5, 2020).

3 Ripley BD. The R project in statistical computing. *MSOR Connections The newsletter of the LTSN Maths* 2001. http://citeseerx.ist.psu.edu/viewdoc/download?doi=10.1.1.430.3979&rep=rep1&type=pdf.

4 ISO/IEC 9075-1:2016. 2018. https://www.iso.org/standard/63555.html (accessed Nov 5, 2020).

5 Java Software. http://oracle.com/java (accessed Dec 20, 2020).

6 Apache Spark™ - Unified Analytics Engine for Big Data. https://spark.apache.org/ (accessed Nov 5, 2020).

7 Fleurence RL, Curtis LH, Califf RM, Platt R, Selby JV, Brown JS. Launching PCORnet, a national patient-centered clinical research network. *J Am Med Inform Assoc* 2014; **21**: 578–82.

8 FitzHenry F, Resnic FS, Robbins SL, *et al.* Creating a Common Data Model for Comparative Effectiveness with the Observational Medical Outcomes Partnership. *Appl Clin Inform* 2015; **6**: 536–47.

9 Visweswaran S, Becich MJ, D'Itri VS, *et al.* Accrual to Clinical Trials (ACT): A Clinical and Translational Science Award Consortium Network. *JAMIA Open* 2018; **1**: 147–52.

10 Topaloglu U, Palchuk MB. Using a Federated Network of Real-World Data to Optimize Clinical Trials Operations. *JCO Clin Cancer Inform* 2018; **2**: 1–10.

11 Haendel M, Chute C, Gersing K. The National COVID Cohort Collaborative (N3C): Rationale, Design, Infrastructure, and Deployment. *J Am Med Inform Assoc* 2020; published online Aug 17. DOI:10.1093/jamia/ocaa196.

12 Quan H, Li B, Couris CM, *et al.* Updating and validating the Charlson comorbidity index and score for risk adjustment in hospital discharge abstracts using data from 6 countries. *Am J Epidemiol* 2011; **173**: 676–82.

13 Wasey, JO Lang, M and R Core Team. icd: Comorbidity Calculations and Tools for ICD-9 and ICD-10 Codes. 2020; published online May 31. https://rdrr.io/cran/icd/ (accessed Dec 18, 2020).

14 WHO. Anatomical Therapeutic Chemical (ATC) classification. https://www.whocc.no/atc_ddd_index_and_guidelines/atc_ddd_index/ (accessed Dec 15, 2020).

15 Venkataraman S, Yang Z, Liu D, *et al.* SparkR: Scaling R Programs with Spark. In: Proceedings of the 2016 International Conference on Management of Data. New York, NY, USA: Association for Computing Machinery, 2016: 1099–104.

16 pyspark package — PySpark 3.0.1 documentation. https://spark.apache.org/docs/latest/api/python/pyspark.html (accessed Dec 18, 2020).

17 Pedregosa F, Varoquaux G, Gramfort A, *et al.* Scikit-learn: Machine Learning in Python. *J Mach Learn Res* 2011; **12**: 2825–30.

18 Create Elegant Data Visualisations Using the Grammar of Graphics. https://ggplot2.tidyverse.org/index.html (accessed Nov 5, 2020).

19 Brunson J. ggalluvial: Layered Grammar for Alluvial Plots. *J Open Source Softw* 2020; **5**: 2017.

© 2021 Bennett TD et al. *JAMA Network Open.*

20 Campitelli E. ggnewscale: Multiple Fill and Colour Scales in ‘ggplot2’. R package version 0.4.4. 2020; published online Dec 2. https://CRAN.R-project.org/package=ggnewscale (accessed Dec 18, 2020).

21 Hunter JD. Matplotlib: A 2D Graphics Environment. *Computing in Science Engineering* 2007; **9**: 90–5.

22 Phenotype_Data_Acquisition. Github https://github.com/National-COVID-Cohort-Collaborative/Phenotype_Data_Acquisition (accessed Nov 5, 2020).

23 Hripcsak G, Duke JD, Shah NH, *et al.* Observational Health Data Sciences and Informatics (OHDSI): Opportunities for Observational Researchers. *Stud Health Technol Inform* 2015; **216**: 574–8.

24 WHO Working Group on the Clinical Characterisation and Management of COVID-19 infection. A minimal common outcome measure set for COVID-19 clinical research. *Lancet Infect Dis* 2020; **20**: e192–7.

25 Hripcsak G, Ryan PB, Duke JD, *et al.* Characterizing treatment pathways at scale using the OHDSI network. *Proc Natl Acad Sci U S A* 2016; **113**: 7329–36.

© 2021 Bennett TD et al. *JAMA Network Open.*
