# Supplementary material for: Clinical Characterization and Prediction of Clinical Severity of SARS-CoV-2 Infection Among US Adults Using Data From the US National COVID Cohort Collaborative
Source: JAMA Netw Open. 2021 Jul 13;4(7):e2116901. doi: 10.1001/jamanetworkopen.2021.16901 (PMC8278272; doi:10.1001/jamanetworkopen.2021.16901)
Supplement: Supplement 2. — The National COVID Cohort Collaborative (N3C) Consortium Members [file jamanetwopen-e2116901-s002.pdf]

Supplementary Online Material: Nonauthor Collaborators

*Group Name(s): The National COVID Cohort Collaborative (N3C) Consortium

*First Name and Middle Initial(s)	*Last Name	*Suffix (eg, Jr, III)	Academic Degrees	Institution
Joel	Gagnier		BA, MS, PhD, ND	The University of Michigan at Ann Arbor
Siqing	Hu		BS, MS	The University of Michigan at Ann Arbor
Kanchan	Lota		MPH	The University of Michigan at Ann Arbor
Sarah	Maidlow		AA	The University of Michigan at Ann Arbor
David A.	Hanauer		MS, MD	University of Michigan
Kevin	Weatherwax		CCRC, CCRA	University of Michigan–Ann Arbor
Nikhila	Gandrakota		MPH, MBBS	Emory University
Rishikesan	Kamaleswaran		PhD	Emory University School of Medicine
Greg	Martin		MS, MD	Emory University
Jingjing	Qian		PhD	Auburn University, Auburn, AL
Jason E.	Farley		MPH, PhD, RN, NP	School of Nursing, Johns Hopkins University, Baltimore, MD USA
Patricia A.	Francis		MS, MA	Johns Hopkins University School of Medicine
Dazhi	Jiao		MS	Johns Hopkins University School of Medicine

Hadi	Kharrazi	PhD, MD	Johns Hopkins School of Public Health
Justin T.	Reese	PhD	Environmental Genomics and Systems Biology Division, Lawrence Berkeley National Laboratory
Mariam	Deacy	MD	National Center for Advancing Translational Sciences, National Institutes of Health
Usman	Sheikh	MBA	National Center for Advancing Translational Sciences, National Institutes of Health
Jake	Chen	PhD	University of Alabama at Birmingham
Michael	Patton	BA	University of Alabama at Birmingham
Thomas	Ramsey	PhD	University of Alabama at Birmingham
Jasvinder	Singh	MS, MBBS	University of Alabama at Birmingham
James J.	Cimino	MD	University of Alabama-Birmingham
Jing	Su	PhD	Indiana University
William G	Adams	MD	Boston University, /Boston Medical Center, Boston, MA, USA
Tim	Duong	PhD	Albert Einstein College of Medicine, Bronx, NY, USA

John B.	Buse	MD, PhD	University of North Carolina School of Medicine
Jessica Yasmine	Islam	MPH, PhD	University of North Carolina at Chapel Hill
Jihad S.	Obeid	MD	Department of Public Health Sciences, Medical University of South Carolina, Charleston, SC, USA
Stephane	Meystre	PhD, MD	Medical University of South Carolina, Charleston, SC, USA
Steve	Patterson	BS	University of Virginia
Misha	Zemmel	MS	University of Virginia
Ron	Grider	BS	University of Virginia, Charlottesville, VA, USA
Ashley	Martinez	MS, PhD, PharmD	Rush University Medical Center
Carlos	Santos	MD, MPHS	Rush University Medical Center
Julian	Solway	MD	University of Chicago
Ryan	Chiu	BS	University of Illinois at Chicago
Gerald B.	Brown	NA	University of Texas Medical Branch at Galveston
Jiafeng	Cui	BS	University of Texas Medical Branch at Galveston

Sharon	Liang	MS, MD	University of Texas Medical Branch at Galveston
Kamil	Khanipov	BS, PhD	University of Texas Medical Branch
Jeremy Richard	Harper	MS	Owl Health Works LLC, Indianapolis, IN, USA
Peter J.	Embi	MS, MD	Regenstrief Institute, Indianapolis, IN, USA
David A.	Eichmann	BS, MS, PhD	School of Library and Information Science, The University of Iowa
Boyd M.	Knosp	MS	University of Iowa Institute for Clinical and Translational Science, The University of Iowa
William	Hillegass	MPH, PhD, MD	University of Mississippi Medical Center, Jackson, MS, USA
Chunlei	Wu	PhD	Department of Integrative Structural and Computational Biology, The Scripps Research Institute, La Jolla, CA, USA
James	Aaron	MHA	University of Kentucky
Darren	Henderson	BS	University of Kentucky
Muhammad	Gul	MD	University of Kentucky

Tamela	Harper	MHA	University of Kentucky
Daniel	Harris	PhD	University of Kentucky
Jeff	Talbert	PhD	University of Kentucky
Neil	Bahroos	MS, MBA	University of Southern California, Los Angeles, CA, USA
Steven	Dubinett	MD	University of California, Los Angeles, Los Angeles, CA, USA
Jomol	Mathew	PhD	University of Wisconsin-Madison
Gabriel	McMahan	BS	University of Wisconsin-Madison, Madison, WI, USA
Hongfang	Liu	PhD	Mayo Clinic, Rochester, MN, USA
Claudia	Lucchinetti	MD	Mayo Clinic, Rochester, MN, USA
David L	Schwartz	MD	The University of Tennessee Health Science Center, Memphis, TN, USA
Ralph	Sacco	BS, MS, MD	University of Miami
Peyman	Taghioff	MS	University of Miami
Diane	Harper	MS, MPH, MD	Michigan Medicine
Denise	Angst	PhD, RN	Aurora Health, Milwaukee, WI, USA
Andrew	Marek	BBA	Aurora Health, Milwaukee, WI, USA

Carlos E.	Figueroa Castro		MS, MD	Medical College of Wisconsin, Milwaukee, WI, USA
Bruce	Blazar		MD	University of Minnesota, Minneapolis, MN, USA
Steven G.	Johnson		PhD	University of Minnesota, Minneapolis, MN, USA
Melissa	Basford		MBA	Vanderbilt University Medical Center
Laura	Jones		MPH	Vanderbilt University Medical Center
Gordon	Bernard		BS, MD	Vanderbilt University Medical Center, Nashville, TN, USA
Rosalind	Wright		MPH, MD	Icahn School of Medicine at Mount Sinai
Joseph	Finkelstein		PhD, MD	Icahn School of Medicine at Mount Sinai, New York, NY, USA
Thomas R.	Campion	Jr.	MS, PhD	Weill Cornell Medicine, Cornell University, New York, NY, USA
Christopher E.	Mason		PhD	Weill Cornell Medicine, Cornell University, New York, NY, USA
Xiaobo	Fuld		MS	Weill Cornell Medicine
Alfred	Anzalone		MS	University of Nebraska Medical Center, Omaha, NE, USA

James C.	McClay	MD	University of Nebraska Medical Center, Omaha, NE, USA
Shyam	Visweswaran	PhD, MD	Department of Biomedical Informatics, University of Pittsburgh
Connor	Cook	MA	Oregon Clinical and Translational Research Institute, Oregon Health & Science University
Alexandra	Dest	MPH	Oregon Health & Science University
David	Ellison	BS, MD	Oregon Health & Science University
Rose	Relevo	BA	Oregon Health & Science University
Andrea M.	Volz	BS	Oregon Clinical and Translational Research Institute, Oregon Health & Science University
Chengda	Zhang	MD	Oregon Health & Science University
Martha	Tenzer	BA	Carilion Clinic
David S.	Bowers	BA	Carilion Clinic, Roanoke, VA, USA
Francis	Farrell	PhD	Carilion Clinic, Roanoke, VA, USA
Qiuyuan	Qin	MS, PhD	University of Rochester Medical Center
Martin	Zand	PhD, MD	University of Rochester Medical Center
Jeanne	Holden-Wiltse	MPH, MBA	University of Rochester

Ramkiran	Gouripeddi	MS, MD	University of Utah, Salt Lake City, UT, USA
Julio	Facelli	PhD	University of Utah
Robert	Clark	MD	University of Texas Health Science Center at San Antonio, San Antonio, TX, USA
Benjamin	Becerra	PhD, MD	California State University, San Bernardino, San Bernadino, CA, USA
Yao	Yan	BS	Molecular Engineering & Sciences Institute, University of Washington, Seattle
Jimmy	Phuong	PhD	School of Medicine, Division of Biomedical and Health Informatics, University of Washington
Yooree	Chae	MS	Sage Bionetworks
Rena C.	Patel	MPH, MD, MPhil	University of Washington
Christine	Suver	PhD	Sage Bionetworks
Elizabeth	Zampino	MS	University of Washington
Ahmed S.	Said	PhD, MD	Washington University in St. Louis, St. Louis, MO, USA
Philip R.O.	Payne	PhD	Institute for Informatics, Washington University in St. Louis, Saint Louis, MO, USA
Randeep	Jawa	MD	Stony Brook Medicine

Peter L.	Elkin	MD	Department of Biomedical Informatics, University at Buffalo, Buffalo, NY, USA
Farrukh M	Koraishy	PhD, MD	Stony Brook University
George	Golovko	PhD	The University of Texas Medical Branch
Vignesh	Subbian	MS, PhD	College of Engineering, The University of Arizona, Tucson, AZ, USA
Daniel	Weisdorf	PhD, MD	University of Minnesota
Lawrence	Sinoway	PhD	Penn State University, University Park, PA, USA
Hiroki	Morizono	BS, PhD	Children's National Hospital, Washington, DC, USA
Keith A.	Crandall	PhD, MA	Computational Biology Institute and Department of Biostatistics & Bioinformatics, Milken Institute School of Public Health, The George Washington University, Washington, DC, USA

Ali	Rahnavard	PhD	Computational Biology Institute and Department of Biostatistics & Bioinformatics, Milken Institute School of Public Health, The George Washington University, Washington, DC, USA
Nawar	Shara	PhD	Georgetown University, Washington, DC, USA
Alysha	Taxter	MD, MSCE	Wake Forest University Health Sciences
Brian	Ostasiewski	BS	Wake Forest University Health Sciences, Winston Salem, NC, USA
Qianqian	Song	PhD	Department of Cancer Biology, Wake Forest School of Medicine, Winston-Salem, NC, USA
Uma Maheswara Reddy	Vangala	MS	University of Massachusetts Medical School
Katherine Ruiz	De Luzuriaga	MD	University of Massachusetts Medical School Worcester
Rasha	Khatib	MS, PhD	Advocate Health Care Network

John	Kirwan	MS, PhD	Pennington Biomedical Research Center
James	von Oehsen	BS, PhD	Rutgers, The State University of New Jersey
Jason	Moore	PhD	University of Pennsylvania
Ankit	Sakhuja	MD	West Virginia University
Joni	Rutter	PhD	National Center for Advancing Translational Sciences, National Institutes of Health

Location (city, state/province, country)	Role or Contribution, eg, chair, principal investigator	Group (if more than 1 Group listed in the byline) and/or Subgroup (eg, Steering Committee)
Ann Arbor, MI, USA	clinical subject matter expertise, manuscript drafting	
Ann Arbor, MI, USA	clinical data model expertise, data analysis, data curation, data integration, data quality assurance, database / information systems admin	
Ann Arbor, MI, USA	project management	
Ann Arbor, MI, USA	data analysis, data curation, data integration, data quality assurance, data security, database / information systems admin, project management, regulatory oversight / admin	
Ann Arbor, MI, USA	data curation, data integration, manuscript review and editing	
Ann Arbor, MI, USA	funding acquisition, governance, project evaluation, regulatory oversight / admin	
Atlanta, GA, USA	manuscript review and editing	
Atlanta, GA, USA	data analysis, data integration, data quality assurance	
Atlanta, GA, USA	clinical subject matter expertise, manuscript review and editing	
Auburn, AL	clinical subject matter expertise, clinical data model expertise	
Baltimore, MD USA	clinical subject matter expertise, project management	
Baltimore, MD, USA	project management	
Baltimore, MD, USA	data curation, data quality assurance	

Baltimore, MD, USA	clinical subject matter expertise, data analysis, manuscript review and editing, N3C Phenotype definition
Berkeley, CA, USA	biological subject matter expertise, data analysis, statistical analysis
Bethesda, MD, USA	software engineering (of enclave platform)
Bethesda, MD, USA	data analysis, data quality assurance, project management
Birmingham, AL, USA	biological subject matter expertise, data analysis, database / information systems admin, statistical analysis
Birmingham, AL, USA	biological subject matter expertise, clinical subject matter expertise
Birmingham, AL, USA	manuscript drafting, manuscript review and editing, statistical analysis
Birmingham, AL, USA	clinical subject matter expertise, manuscript review and editing
Birmingham, AL, USA	data curation, manuscript review and editing
Bloomington, IN, USA	clinical data model expertise, clinical subject matter expertise, data curation
Boston, MA, USA	clinical subject matter expertise, data curation, data integration
Bronx, NY, USA	data curation, data quality assurance, database / information systems admin, manuscript review and editing, project management

Chapel Hill, NC, USA	clinical subject matter expertise, marketing and communications, regulatory oversight / admin
Chapel Hill, NC, USA	manuscript review and editing, project management
Charleston, SC, USA	clinical subject matter expertise, data curation, manuscript review and editing, marketing and communications, regulatory oversight / admin
Charleston, SC, USA	data integration, funding acquisition, marketing and communications, project management, regulatory oversight / admin
Charlottesville, VA, USA	data integration, database / information systems admin
Charlottesville, VA, USA	data integration, database / information systems admin
Charlottesville, VA, USA	data integration, database / information systems admin
Chicago, IL, USA	data curation, database / information systems admin
Chicago, IL, USA	clinical data model expertise, clinical subject matter expertise, data curation, data quality assurance, database / information systems admin
Chicago, IL, USA	governance
Chicago, IL, USA	database / information systems admin
Galveston, Galveston, TX, USA	project management
Galveston, Galveston, TX, USA	clinical data model expertise, data integration, data quality assurance, database / information systems admin, software engineering (of enclave platform)

Galveston, Galveston, TX, USA	biological subject matter expertise, clinical data model expertise, clinical subject matter expertise, data analysis, data curation, data integration, data quality assurance, data security, data visualization, database / information systems admin, N3C Phenotype definition
Galveston, TX, USA	data curation, data integration, data quality assurance, project management, regulatory oversight / admin
Indianapolis, IN, USA	clinical data model expertise, data analysis, governance, N3C Phenotype definition
Indianapolis, IN, USA	data curation, data quality assurance, funding acquisition, governance, manuscript review and editing
Iowa City, IA, USA	data visualization, database / information systems admin, funding acquisition, project management, software engineering (of enclave platform)
Iowa City, IA, USA	data curation
Jackson, MS, USA	clinical data model expertise, clinical subject matter expertise, data curation
La Jolla, CA, USA	data integration
Lexington, KY, USA	governance, project management
Lexington, KY, USA	data integration, data quality assurance, database / information systems admin, N3C Phenotype definition
Lexington, KY, USA	clinical subject matter expertise

Lexington, KY, USA	data security, governance, regulatory oversight / admin
Lexington, KY, USA	clinical data model expertise, data curation, data integration, data quality assurance, database / information systems admin
Lexington, KY, USA	manuscript review and editing
Los Angeles, CA, USA	clinical data model expertise, data quality assurance, database / information systems admin, N3C Phenotype definition
Los Angeles, Los Angeles, CA, USA	funding acquisition, governance
Madison, WI, USA	data integration
Madison, WI, USA	clinical data model expertise, data curation
Mayo Clinic, Rochester, MN, USA	data integration, N3C Phenotype definition
Mayo Clinic, Rochester, MN, USA	data analysis
Memphis, TN, USA	project management
Miami, FL, USA	governance
Miami, FL, USA	data analysis, data curation, data quality assurance, data security, database / information systems admin
Michigan Medicine	biological subject matter expertise, clinical subject matter expertise, manuscript review and editing
Milwaukee, WI, USA	project management
Milwaukee, WI, USA	data curation

Milwaukee, WI, USA	
Minneapolis, MN, USA	funding acquisition
Minneapolis, MN, USA	data quality assurance, N3C Phenotype definition
Nashville, TN, USA	data integration, project management
Nashville, TN, USA	clinical data model expertise, data curation, data quality assurance, project management, regulatory oversight / admin
Nashville, TN, USA	database / information systems admin, funding acquisition, governance, manuscript review and editing
New York, NY, USA	data curation, data integration, funding acquisition, manuscript review and editing
New York, NY, USA	biological subject matter expertise, clinical data model expertise, clinical subject matter expertise, data curation, data integration, data quality assurance, data security, funding acquisition, project management, regulatory oversight / admin
New York, NY, USA	data integration, project management, regulatory oversight / admin
New York, NY, USA	biological subject matter expertise, clinical subject matter expertise
New York, NY, USA	data curation, data integration, data quality assurance, data security, database / information systems admin
Omaha, NE, USA	N3C Phenotype definition, project evaluation

Omaha, NE, USA	clinical subject matter expertise, funding acquisition, project management
Pittsburgh, PA, USA	clinical data model expertise, N3C Phenotype definition
Portland, OR, USA	project management, regulatory oversight / admin
Portland, OR, USA	project evaluation
Portland, OR, USA	clinical subject matter expertise, funding acquisition, manuscript review and editing
Portland, OR, USA	manuscript review and editing
Portland, OR, USA	project management
Portland, OR, USA	clinical subject matter expertise
Roanoke, VA, USA	database / information systems admin
Roanoke, VA, USA	database / information systems admin
Roanoke, VA, USA	project management
Rochester, NY, USA	data visualization
Rochester, NY, USA	funding acquisition, governance, regulatory oversight / admin
Rochester, NY, USA	database / information systems admin, project management

Salt Lake City, UT, USA	database / information systems admin, manuscript review and editing
Salt Lake City, Utah, USA	data integration, funding acquisition, project management
San Antonio, San Antonio, TX, USA	
San Bernadino, CA, USA	data curation, data integration, data quality assurance, governance
Seattle, WA, USA	clinical data model expertise, manuscript review and editing
Seattle, WA, USA	data integration
Seattle, WA, USA	project management
Seattle, WA, USA	clinical subject matter expertise
Seattle, WA, USA	governance
Seattle, WA, USA	project evaluation, project management, regulatory oversight / admin
St. Louis, MO, USA	manuscript review and editing
St. Louis, Saint Louis, MO, USA	clinical data model expertise, data curation, data integration, funding acquisition, manuscript review and editing, N3C Phenotype definition, project management
Stony Brook Medicine	clinical subject matter expertise, N3C Phenotype definition

Stony Brook, NY, USA	clinical data model expertise, data curation, data integration, governance, N3C Phenotype definition
Stony Brook, NY, USA	manuscript review and editing, N3C Phenotype definition
The Galveston, TX, USA	data curation, data integration, data quality assurance, database / information systems admin, governance, project management, regulatory oversight / admin
Tucson, AZ, USA	clinical data model expertise, data quality assurance, manuscript drafting, project management
University of Minnesota	funding acquisition
University Park, PA, USA	
Washington, DC, USA	data curation, data integration, database / information systems admin
Washington, DC, USA	biological subject matter expertise, data integration, funding acquisition, manuscript review and editing, regulatory oversight / admin

Washington, DC, USA	biological subject matter expertise, clinical data model expertise, clinical subject matter expertise, data analysis, funding acquisition, manuscript review and editing, statistical analysis
Washington, DC, USA	data integration
Winston Salem, NC, USA	regulatory oversight / admin
Winston Salem, NC, USA	data curation, data integration, data quality assurance
Winston-Salem, NC, USA	manuscript review and editing
Worcester, MA, USA	clinical data model expertise, clinical subject matter expertise, data analysis, data curation, data integration, data quality assurance, data security, data visualization, database / information systems admin, marketing and communications, project evaluation, project management, software engineering (of enclave platform)
Worcester, MA, USA	clinical data model expertise, clinical subject matter expertise, governance, marketing and communications, project evaluation, project management, regulatory oversight / admin
Downers Grove, IL, USA	clinical data model expertise, data integration, project management

Baton Rouge, LA, USA	biological subject matter expertise, clinical subject matter expertise, data quality assurance, regulatory oversight / admin
Brunswick, NJ, USA	data analysis, data integration, data quality assurance, data security, governance
Philadelphia, PA, USA	manuscript review and editing, marketing and communications
Morgantown, WV, USA	clinical subject matter expertise, manuscript review and editing
Bethesda, MD, USA	governance
